# Successful Implementation of a Medical Student Postpartum Follow-up Phone Call Project

**DOI:** 10.15766/mep_2374-8265.11109

**Published:** 2021-02-19

**Authors:** Cynthia Abraham

**Affiliations:** 1 Assistant Professor, Department of Obstetrics, Gynecology, and Reproductive Science, Icahn School of Medicine at Mount Sinai and Mount Sinai Health System

**Keywords:** Obstetrics, Postpartum, Women's Health, OB/GYN, Clinical Teaching/Bedside Teaching, Virtual Learning

## Abstract

**Introduction:**

The American College of Obstetricians and Gynecologists recommends prompt postpartum follow-up. However, 40% of women do not attend postpartum visits. These rates are lower in populations with limited resources. In response, the Department of Obstetrics, Gynecology, and Reproductive Science in the Mount Sinai Health System created a postpartum follow-up phone call project that utilized medical students and was conducted at three health system hospitals from April 6 to May 30, 2020.

**Methods:**

The number of patients contacted by medical students within 72 hours of hospital discharge was recorded. Students at two of the three sites also recorded the number of patients who needed (1) urgent evaluation and subsequent hospital readmission, (2) medications prescribed, and (3) referral for social work services. Students completed questionnaires at the project beginning and end regarding confidence in rendering of postpartum care. Confidence level was based on a 5-point Likert scale (1 = *not confident at all*, 5 = *very confident*).

**Results:**

Nine students participated. Overall, confidence on providing postpartum care significantly increased from 2.2 to 3.7 (*p* < .001). Three hundred eighty-seven patients were contacted. Four patients were advised to return to the hospital emergently; two were readmitted. Forty-seven patients needed medication prescribed. Two patients were referred for social work services.

**Discussion:**

Our medical student–driven postpartum follow-up phone call project was associated with a high number of patients called and management of significant postpartum issues. Students’ confidence in managing postpartum issues was significantly higher after versus before project participation.

## Educational Objectives

By the end of this session, learners will be able, via telephone calls made within 48–72 hours of delivery, to:

1. Manage postpartum pain.
2. Assess abnormal uterine bleeding.
3. Screen for pre-eclampsia.
4. Screen for depression.
5. Counsel on contraception.
6. Counsel on breastfeeding.
7. Provide advice on newborn care.
8. Screen for coronavirus symptoms.
9. Counsel on coronavirus prevention.

## Introduction

On March 11, 2020, the World Health Organization (WHO) declared the spread of coronavirus disease (COVID-19) to be a pandemic. To date, the number of COVID-19 cases in the United States has exceeded those of Italy and China. As of April 20, 2020, the state of New York accounted for more than 30% of nationwide cases, more than any other state.^1^ More than half of New York's cases were in New York City, which had been deemed the epicenter of the COVID-19 pandemic.

On March 16, 2020, the academic year for third-year medical students at the Icahn School of Medicine at Mount Sinai in New York City ended prematurely. This action was in response to the recommendation from the Association of American Medical Colleges to suspend all student activities requiring direct patient contact. The rationale for suspending involvement of medical students in clinical activities was that students were not considered essential personnel. There was concern that student presence on inpatient units would contribute to increased exposure and disease transmission and a decrease in personal productive equipment. As soon as the COVID-19 pandemic was declared, though, students mobilized themselves into teams and volunteered for various projects. Multiple volunteer projects were created, running from coordinating research studies on the disease progression of this relatively unknown entity to being engaged in telemedicine, reaching out to those in times of need, and giving emotional support. In response, the Department of Obstetrics, Gynecology, and Reproductive Science in the Mount Sinai Health System created a postpartum follow-up phone call initiative that was conducted from April 6 to May 29, 2020.

The postpartum period is a sensitive time, one that is replete with drastic life changes for mothers. Keeping oneself physically and mentally healthy during the first weeks after birth is paramount to achieving long-term well-being. According to the American College of Obstetricians and Gynecologists (ACOG), to optimize postpartum care, it is essential that patients follow up with their obstetric providers within 3 weeks of delivery and schedule a comprehensive visit no later than 12 weeks postpartum.^2^ It is imperative that an assessment of the following occur: (1) physical recovery from childbirth, (2) resolution of complications occurring during hospital admission, (3) contraception and family planning, (4) sleep and fatigue, (5) infant care and feeding, (6) depression, (7) management of chronic medical conditions, and (8) health care maintenance. The postpartum period has been called the fourth trimester.

Given the myriad concerns that arise postpartum, the WHO has further recommended that routine postpartum evaluations be made at 3 days, at 1–2 weeks, and at 6 weeks after delivery.^3^ However, 40% of women do not attend their postpartum visits. These rates are even lower in populations with limited access to resources.^2^ These barriers may potentially lead to adverse maternal and neonatal outcomes and serve to highlight health care disparities.^4^ Given lockdown measures in place during the COVID-19 pandemic, it was anticipated that the percentage of women with timely postpartum visits would decrease even further.

The literature on teaching of postpartum care is limited. In a search in *MedEdPORTAL,* both resources found focused on mastery of postpartum depression screening.^5,6^ Neither assessed training on evaluation of any of the following: postpartum pain management, abnormal uterine bleeding, postpartum pre-eclampsia, contraception, breastfeeding, and newborn care.

Therefore, here, the outcomes of an initiative focused on providing postpartum follow-up care utilizing medical students and the effect on medical student knowledge of postpartum issues are presented.

## Methods

Two to three students were assigned to each of three hospitals in the Mount Sinai Health System and made postpartum follow-up phone calls within 72 hours of hospital discharge. Two hospitals were in Manhattan. One hospital was on Long Island. Phone calls were made to only those patients who were delivered by hospitalists. It was anticipated that over the course of 2 months, approximately 500 patients would be called based on the number of deliveries performed by hospitalists at each of the three hospitals.

All third-year medical students participating in the project had to have completed their core clerkship in obstetrics and gynecology (OB/GYN) within the prior 9 months. All students had a virtual 1-hour educational session reviewing scripts they would utilize at the time of the telephone encounters. The slide presentation used during the 1-hour educational session is in Appendix A. The scripts are in Appendices B and C. Students also received a summary of answers to commonly asked questions related to postpartum care, which is in Appendix D. At the termination of the orientation session, students were instructed to rate on a scale of 1–5 (low to high, unfavorable to favorable) the utility of the content and the presentation of the material. Ratings were obtained anonymously.

The scripts outlined the following components of postpartum care that needed to be addressed: (1) pain management, (2) extent of vaginal bleeding and occurrence of signs concerning for postpartum infection, (3) wound appearance, (4) presence of symptoms of postpartum pre-eclampsia in those with a history of hypertension, (5) evaluation of contraceptive options and presence of associated complications if contraception was started immediately postpartum, (6) screening for depression, (7) inquiry on method of infant feeding and rendering of breastfeeding counseling where necessary, and (8) assuring appropriate infant care/follow-up and provision of advice on postcircumcision care, if one had been performed. Given the COVID-19 pandemic, students were further advised to provide counseling on hand hygiene and social distancing. For the subset of patients with coronavirus infection, questions related to fever and respiratory symptoms were also asked in addition to confirming ongoing compliance with anticoagulant administration for those who had undergone cesarean. The scripts used for those without and with coronavirus infection are in Appendices B and C, respectively. Those with coronavirus infection received additional phone calls at 1 week and at 2 weeks after hospital discharge to assess for ongoing presence of coronavirus symptoms and to reinforce recommendations released by the Centers for Disease Control and Prevention. The script for these follow-up phone calls is in Appendix E.

If a patient's primary language was not English, students were required to use the phone interpreter services provided by Pacific Interpreters. Students were not authorized to act as interpreters. All students had experience using Pacific Interpreter's services and were thus comfortable with using them as necessary when engaging in the telephone encounters. Sections in the script had notes regarding answers to questions that necessitated escalation to an attending obstetrician and the associated level of urgency. Attending obstetricians reviewed the answers to the telephone encounters, which were documented electronically. Students used templates in the electronic medical record that corresponded to script content. If indicated, attending obstetricians electronically prescribed medications and made determinations for referral to the emergency department and the labor and delivery unit for urgent evaluation. Referrals were also made to social workers and lactation specialists in the setting of positive depression screens and cases in which breastfeeding difficulties were encountered, respectively. If requested by the attending physician, the student also completed the Edinburgh Depression Scale^7^ in the setting of positive depression screen.

One of the attending obstetricians coordinating the project organized a dedicated didactic curriculum focused on management of postpartum issues. This was performed via videoconferencing. All students participating in the postpartum follow-up phone call project were required to attend all five sessions in order to continue project participation. The didactic curriculum was composed of a series of eight weekly 1-hour lectures that each reviewed one of the following topics: management of normal and abnormal labor, postpartum infection, postoperative complications, hypertension in pregnancy, respiratory infections in pregnancy, contraception, and abnormal uterine bleeding. The lecture learning objectives were to identify pregnancy-related issues and understand how to manage them. It was appreciated that these topics would add a significant amount of context at the time of telephone encounters in light of questions in the script that assessed some of the topics. Moreover, the sessions would provide a refresher pertaining to common obstetric topics that students had not been exposed to since their clerkship in OB/GYN. For all, it had been at least 3 months since they had completed the OB/GYN clerkship. The 11th edition of the *APGO Medical Student Educational Objectives* was used as a guide during lecture construction.^8^

Students completed questionnaires that inquired how confident they were in performing the following: managing pain, assessing abnormal uterine bleeding, screening for pre-eclampsia, counseling on contraception, screening for depression, counseling on breastfeeding, and counseling on newborn care. Students also indicated their overall level of confidence in providing postpartum care. Confidence level was measured on a 5-point Likert scale (1 = *not confident at all,* 3 = *neutral,* 5 = *very confident*). Confidence levels before and after the simulation were compared using *t* tests. A *p* value of less than .05 was considered significant. Students at all three sites where this project was conducted also recorded the number of patients who needed (1) urgent evaluation and subsequent hospital readmission, (2) medications prescribed, and (3) referral for social work services. Resources for access to emergent services and social work referrals were comparable at all three sites.

## Results

Nine medical student learners participated between April 6, 2020, and May 29, 2020. One of the learners was male. Eight were female. All were third-year medical students who had completed their clerkship in OB/GYN within the prior 9 months.

All learners attended the orientation session and reported that they had reviewed the documents that had been sent to them prior to making postpartum follow-up phone calls. All learners reported that the documents they read before making the phone calls were helpful in solidifying their ability to assess and manage postpartum issues. Ratings for the orientation session with regard to the utility of the content and the presentation of the material were 4.5 and 4.6, respectively.

The Table outlines confidence levels before and after involvement in the project pertaining to performance of the following: managing pain, assessing abnormal uterine bleeding, screening for pre-eclampsia, counseling on contraception, screening for depression, counseling on breastfeeding, counseling on newborn care, and overall rendering of postpartum care. Mean learner confidence levels for all parameters significantly increased after involvement in the project (*p* < .05). Overall confidence regarding rendering of care significantly increased from 2.2 to 3.7 (*p* < .01). Confidence level ranges before and after involvement in the project were 1–5.

**Table.**
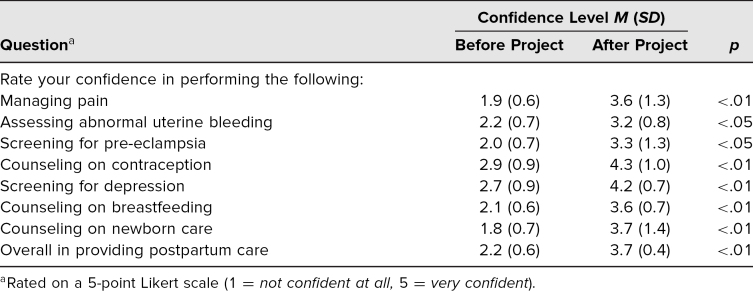
Confidence Levels Before and After Project (*N* = 9)

Attempts were made to call 460 patients; 387 patients responded and had telephone encounters (site 1: 152, site 2: 147, and site 3: 88). At site 1, four patients reported issues that caused them to return to the hospital for urgent evaluation; two were readmitted. At the three sites, a total of 47 patients needed essential medications prescribed. Essential medications were those used to treat pain, elevated blood pressures, and infection. At sites 1 and 3, 20 and 13 patients had coronavirus infection, respectively, and received follow-up phone calls in addition to their initial immediate postpartum follow-up phone call. At site 1, two patients were referred for social work services.

All learners escalated urgent issues, medications to be prescribed, and need for referral to social work promptly. There were no elements of communication noted that were detrimental to patient care; no patient complaints were made to administration.

At the end of the project, all learners reported that it had been immensely valuable as their exposure to postpartum care prior to involvement in the project was limited. Their prior exposure had lasted 2.5 weeks (the duration of half of their third-year core OB/GYN clerkship) and took place months prior to involvement with this project. Based on informal feedback, attending obstetricians who worked with the participating students viewed the task performance of all students favorably.

## Discussion

The advent of mobile applications, wearable devices, text messaging, and live audiovisual communication created the motivation to incorporate technology into medicine. Although telemedicine is currently in its nascent stages, current evidence is promising. Published articles suggest that in the realm of obstetrics, telemedicine has been associated with increased patient satisfaction and has not been linked to an increase in cesarean delivery rates or an increase in the incidence of preterm birth and neonatal intensive care admissions. Additionally, studies evaluating the efficacy of remote monitoring of blood pressures postpartum have shown that this method of surveillance has been linked to a greater adherence to ACOG recommendations pertaining to management of hypertension in the postpartum period. Moreover, text communication associated with web-based platforms has been noted to increase exclusive breastfeeding and breastfeeding continuation rates.^9^

This project was created not only to address postpartum issues during a time in which patients may face a barrier to care but also to educate medical students on the rendering of postpartum care. There was a significant increase in confidence pertaining to overall management of postpartum care from before to after involvement in the project. Across three sites, over a 6-week period, students called 387 patients. Students were responsible for identifying immediate postpartum issues that warranted hospital readmission, mental health issues that required social work assessment, and instances in which patients did not have necessary medications. The project served to alleviate barriers to care that many patients faced. It also attests to the ability to provide patient care in an environment with limited resources. All students indicated a high level of fulfillment during involvement with the project. Students’ satisfaction and increase in confidence level in managing postpartum care highlight the importance of ongoing implementation of this project. All learners indicated that the project was immensely valuable as their postpartum care exposure during the OB/GYN clerkship had been limited, lasting approximately 2 weeks. Additionally, they had been enrolled in the OB/GYN clerkship several months prior to being involved in the project. These findings indicate a need for more exposure and more formal didactics on postpartum care during the third-year OB/GYN clerkship.

One limitation of this study is its primary outcome being confidence level instead of an objective outcome such as retention of knowledge. Future directions could entail assessing knowledge and confidence in relation to management of postpartum issues immediately following the initial educational session and again after several clinical encounters. This would aid in determining effectiveness of the project. Another limitation is the lack of information on patient satisfaction associated with the project. Hence, another direction for study would involve a separate phone call to patients assessing their level of satisfaction with their phone encounter with the student.

In conclusion, the success of the Mount Sinai Health System medical student postpartum follow-up phone call project emphasizes the effectiveness of telemedicine and remote learning. It highlights the increased importance that telemedicine will have in the future, as well as the need for medical education to address this innovative patient care approach. Newer technologies can assist in modifying care to the unique needs of each patient, especially in those populations that have limited access to care. Telemedicine is a platform that can allow one to convey empathy and compassion, qualities that are essential for future physicians to possess. Furthermore, videoconferencing applications will play an augmented role in the dispensing of education in the future. The medical student postpartum follow-up phone call project is an example of our ability to develop contingency plans for addressing gaps in medical education and patient care that may occur in a time of crisis.

## Appendices

Medical Student Postpartum Project.pptxCOVID Negative 72-Hour Follow-up.docxCOVID Positive 72-Hour Follow-up.docxAdditional Guidance.docxCOVID Positive 1- to 2-Week Follow-up.docx
All appendices are peer reviewed as integral parts of the Original Publication.
