# Supplementary material for: Successful Implementation of a Medical Student Postpartum Follow-up Phone Call Project
Source: MedEdPORTAL. 2021 Feb 19;17:11109. doi: 10.15766/mep_2374-8265.11109 (PMC7901253; doi:10.15766/mep_2374-8265.11109)

## Slide 1
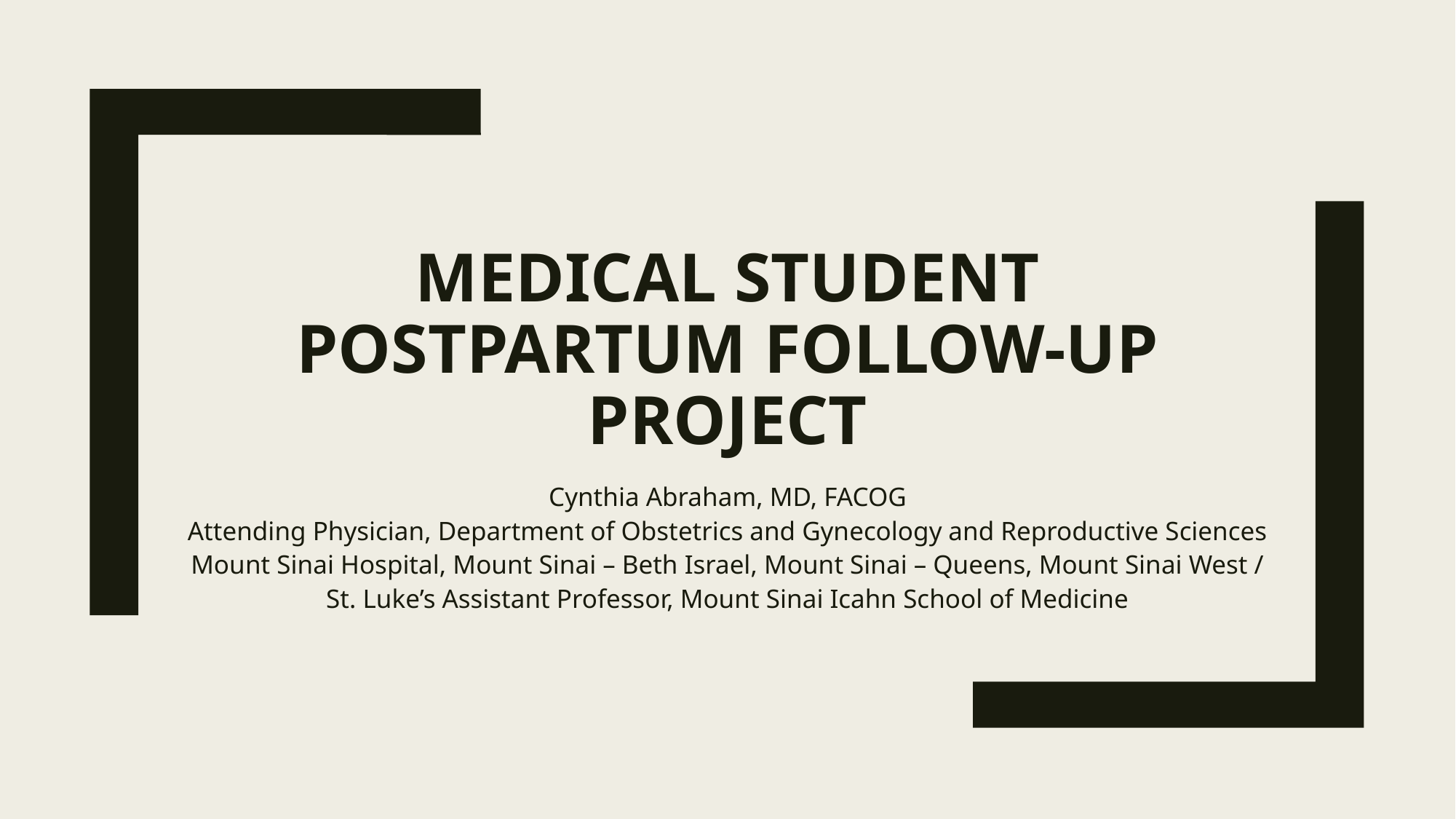

# MEDICAL STUDENT POSTPARTUM FOLLOW-UP PROJECT
Cynthia Abraham, MD, FACOG
Attending Physician, Department of Obstetrics and Gynecology and Reproductive Sciences
Mount Sinai Hospital, Mount Sinai – Beth Israel, Mount Sinai – Queens, Mount Sinai West / St. Luke’s Assistant Professor, Mount Sinai Icahn School of Medicine

## Slide 2
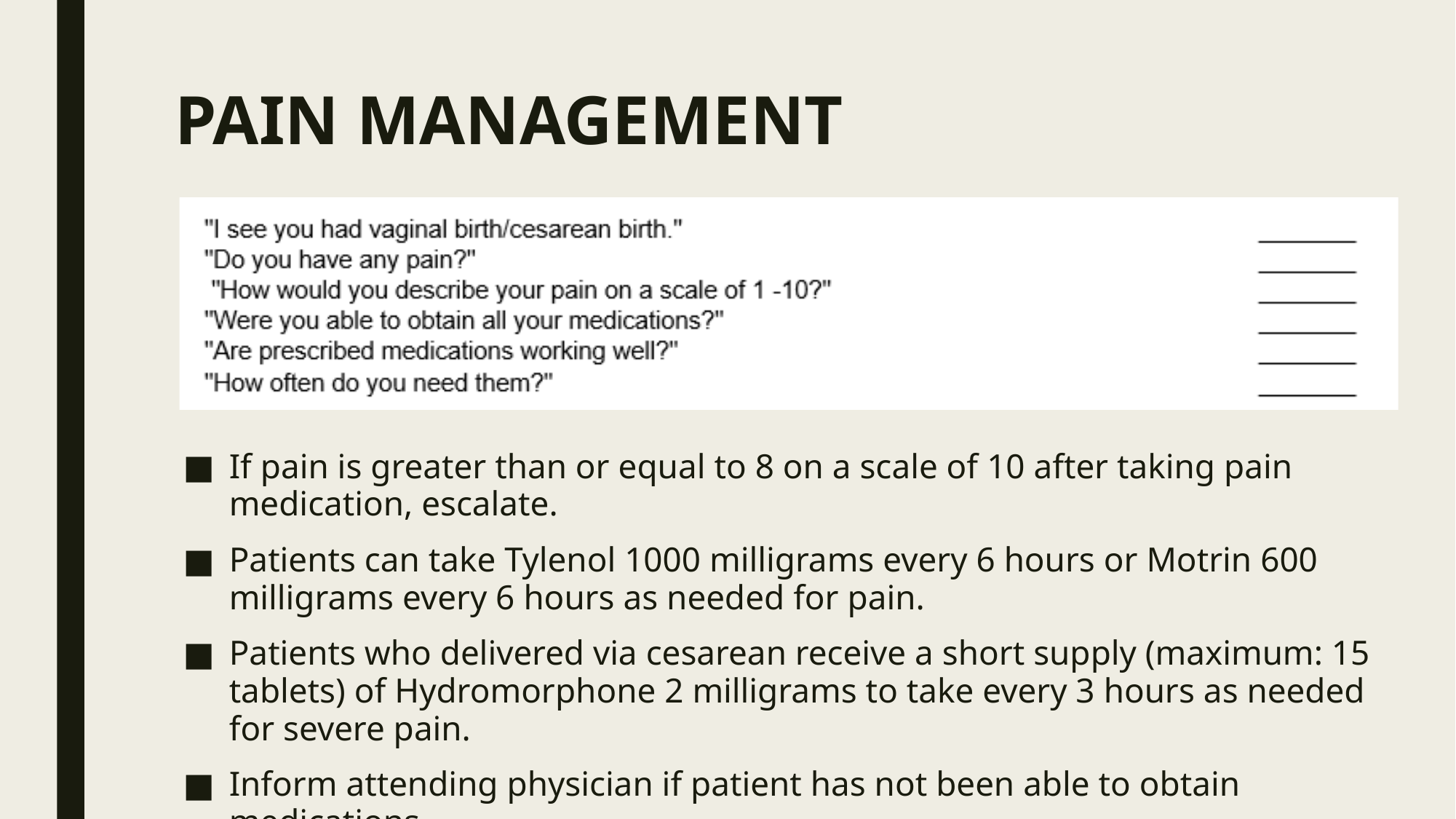

# PAIN MANAGEMENT
If pain is greater than or equal to 8 on a scale of 10 after taking pain medication, escalate.
Patients can take Tylenol 1000 milligrams every 6 hours or Motrin 600 milligrams every 6 hours as needed for pain.
Patients who delivered via cesarean receive a short supply (maximum: 15 tablets) of Hydromorphone 2 milligrams to take every 3 hours as needed for severe pain.
Inform attending physician if patient has not been able to obtain medications.

## Slide 3
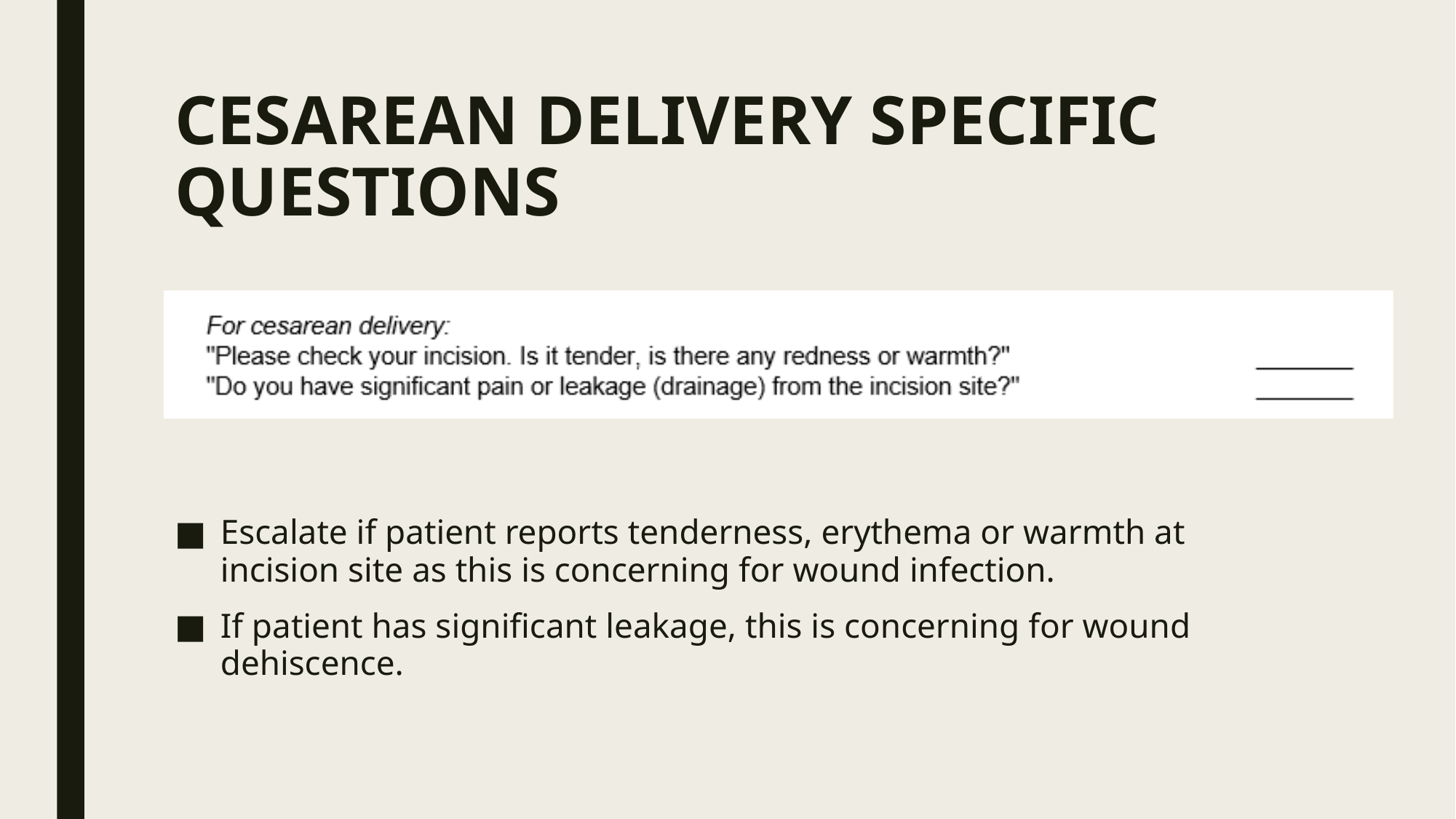

# CESAREAN DELIVERY SPECIFIC QUESTIONS
Escalate if patient reports tenderness, erythema or warmth at incision site as this is concerning for wound infection.
If patient has significant leakage, this is concerning for wound dehiscence.

## Slide 4
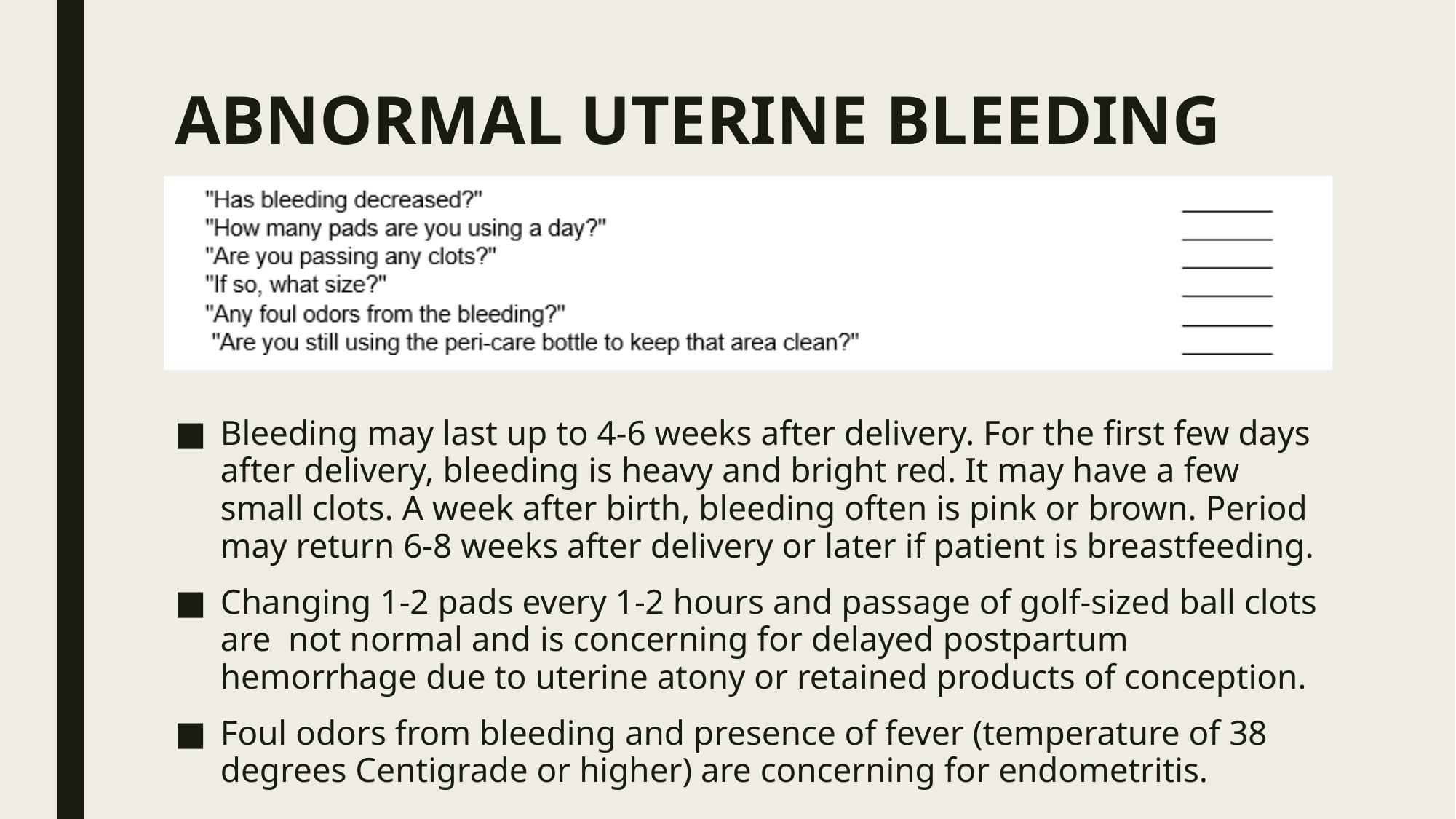

# ABNORMAL UTERINE BLEEDING
Bleeding may last up to 4-6 weeks after delivery. For the first few days after delivery, bleeding is heavy and bright red. It may have a few small clots. A week after birth, bleeding often is pink or brown. Period may return 6-8 weeks after delivery or later if patient is breastfeeding.
Changing 1-2 pads every 1-2 hours and passage of golf-sized ball clots are not normal and is concerning for delayed postpartum hemorrhage due to uterine atony or retained products of conception.
Foul odors from bleeding and presence of fever (temperature of 38 degrees Centigrade or higher) are concerning for endometritis.

## Slide 5
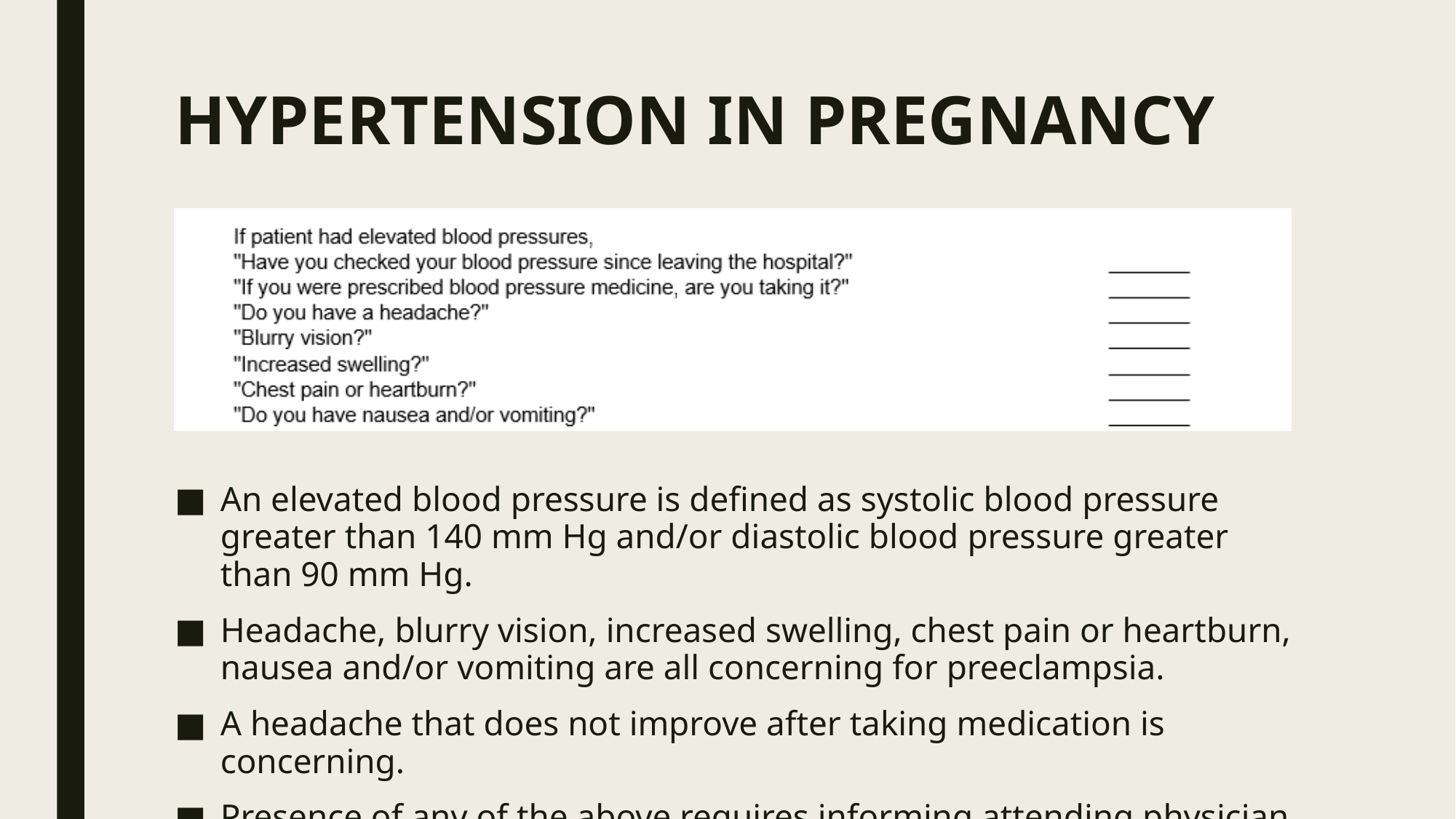

# HYPERTENSION IN PREGNANCY
An elevated blood pressure is defined as systolic blood pressure greater than 140 mm Hg and/or diastolic blood pressure greater than 90 mm Hg.
Headache, blurry vision, increased swelling, chest pain or heartburn, nausea and/or vomiting are all concerning for preeclampsia.
A headache that does not improve after taking medication is concerning.
Presence of any of the above requires informing attending physician.

## Slide 6
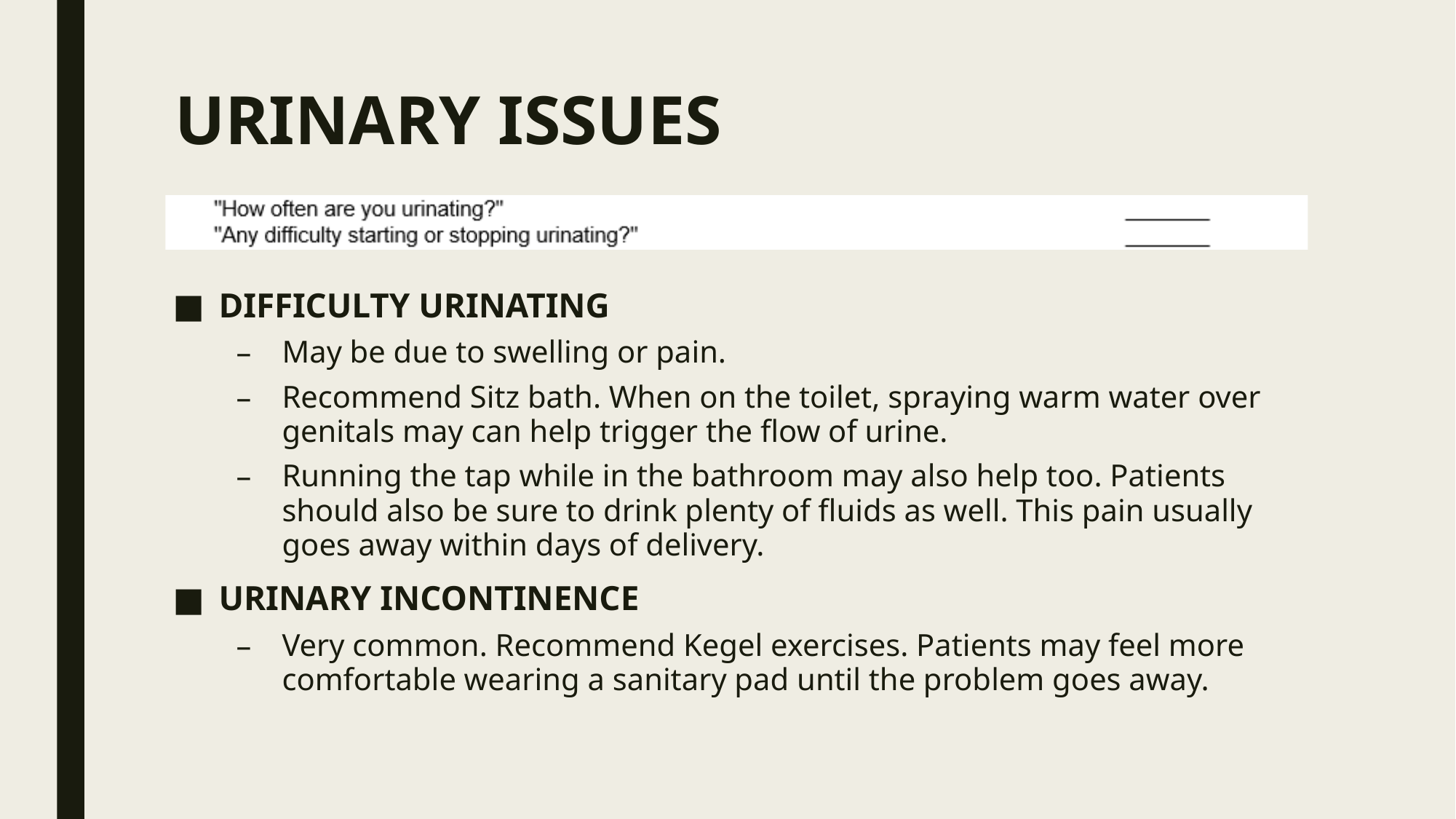

# URINARY ISSUES
DIFFICULTY URINATING
May be due to swelling or pain.
Recommend Sitz bath. When on the toilet, spraying warm water over genitals may can help trigger the flow of urine.
Running the tap while in the bathroom may also help too. Patients should also be sure to drink plenty of fluids as well. This pain usually goes away within days of delivery.
URINARY INCONTINENCE
Very common. Recommend Kegel exercises. Patients may feel more comfortable wearing a sanitary pad until the problem goes away.

## Slide 7
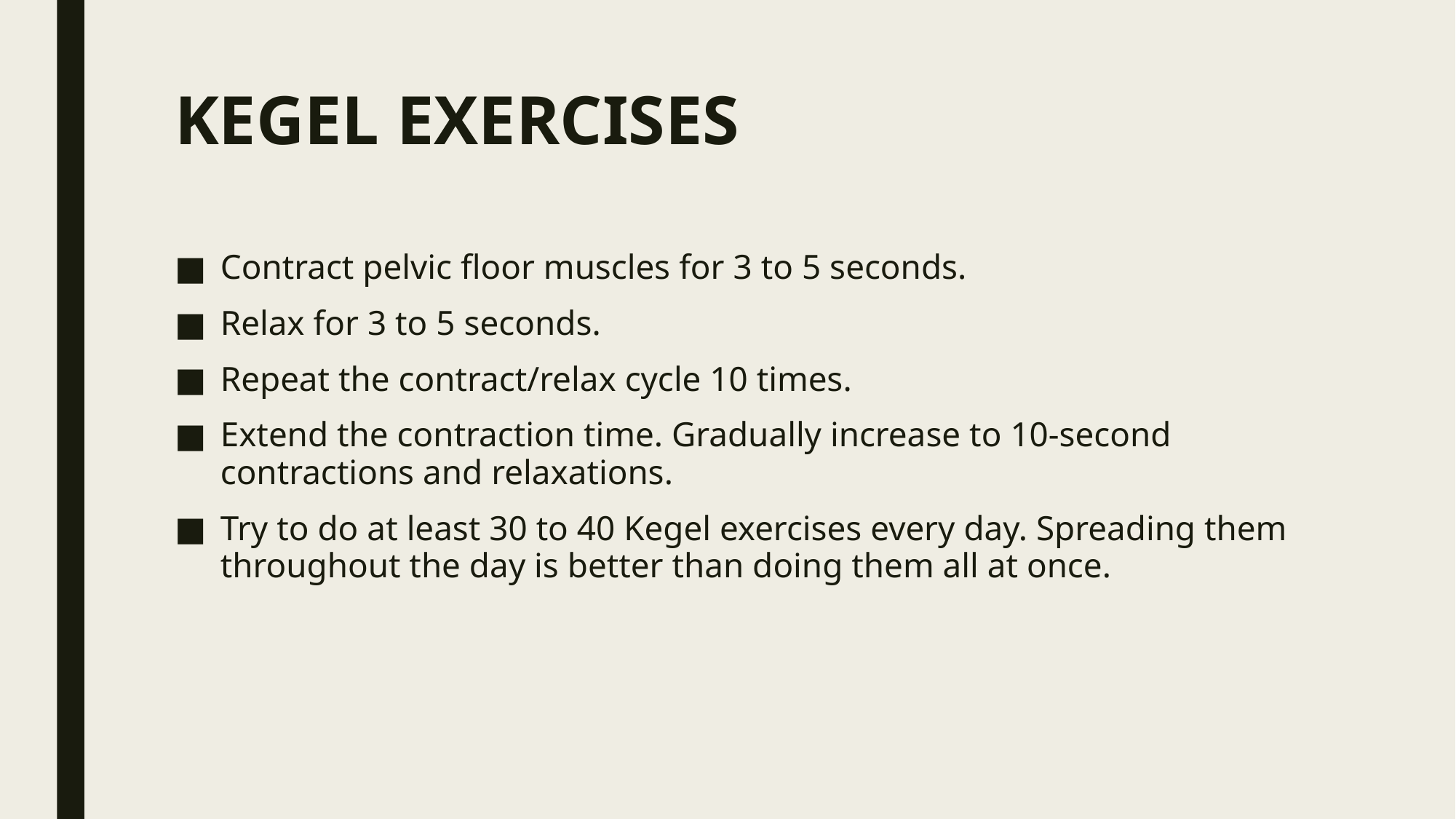

# KEGEL EXERCISES
Contract pelvic floor muscles for 3 to 5 seconds.
Relax for 3 to 5 seconds.
Repeat the contract/relax cycle 10 times.
Extend the contraction time. Gradually increase to 10-second contractions and relaxations.
Try to do at least 30 to 40 Kegel exercises every day. Spreading them throughout the day is better than doing them all at once.

## Slide 8
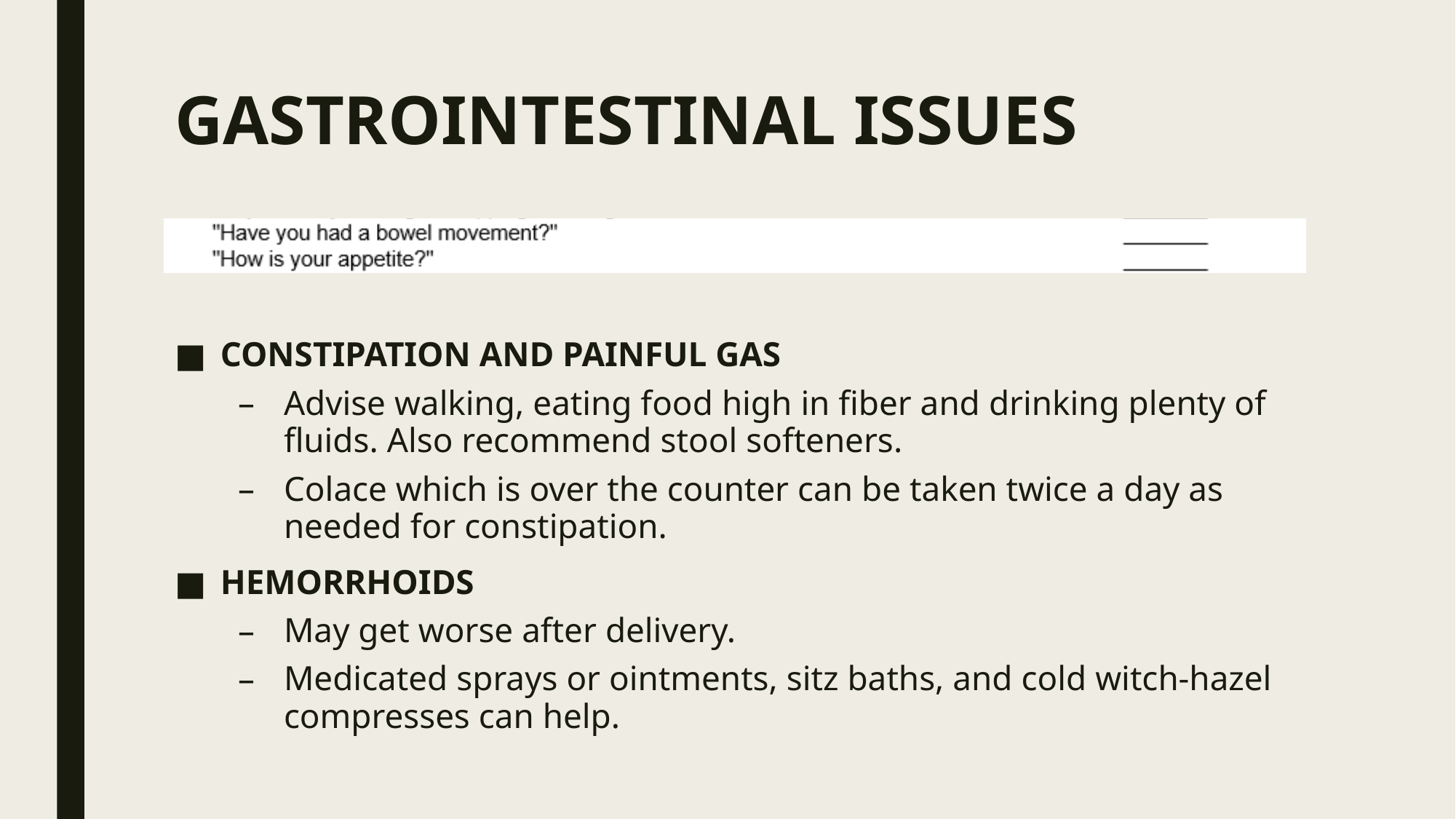

# GASTROINTESTINAL ISSUES
CONSTIPATION AND PAINFUL GAS
Advise walking, eating food high in fiber and drinking plenty of fluids. Also recommend stool softeners.
Colace which is over the counter can be taken twice a day as needed for constipation.
HEMORRHOIDS
May get worse after delivery.
Medicated sprays or ointments, sitz baths, and cold witch-hazel compresses can help.

## Slide 9
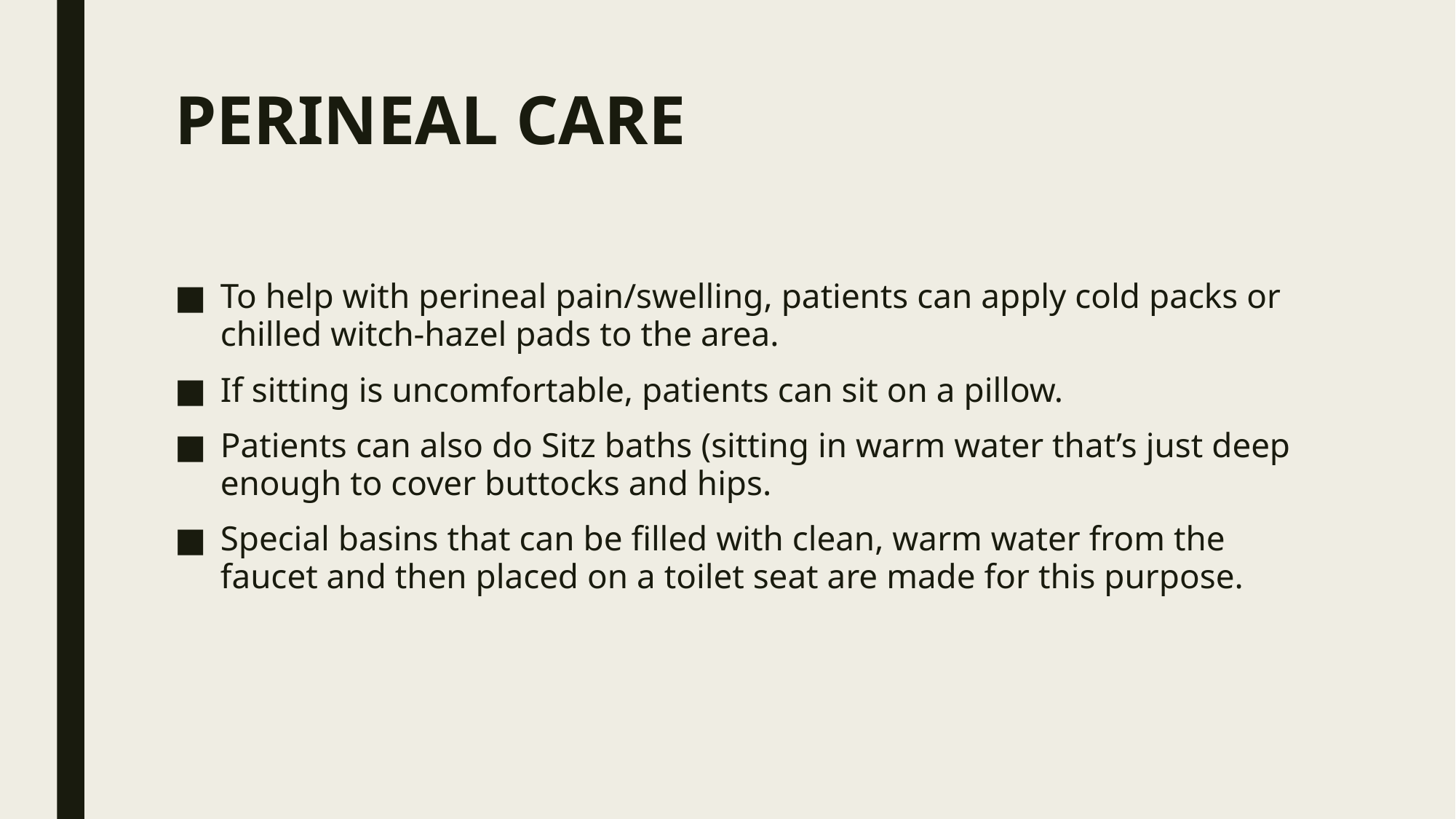

# PERINEAL CARE
To help with perineal pain/swelling, patients can apply cold packs or chilled witch-hazel pads to the area.
If sitting is uncomfortable, patients can sit on a pillow.
Patients can also do Sitz baths (sitting in warm water that’s just deep enough to cover buttocks and hips.
Special basins that can be filled with clean, warm water from the faucet and then placed on a toilet seat are made for this purpose.

## Slide 10
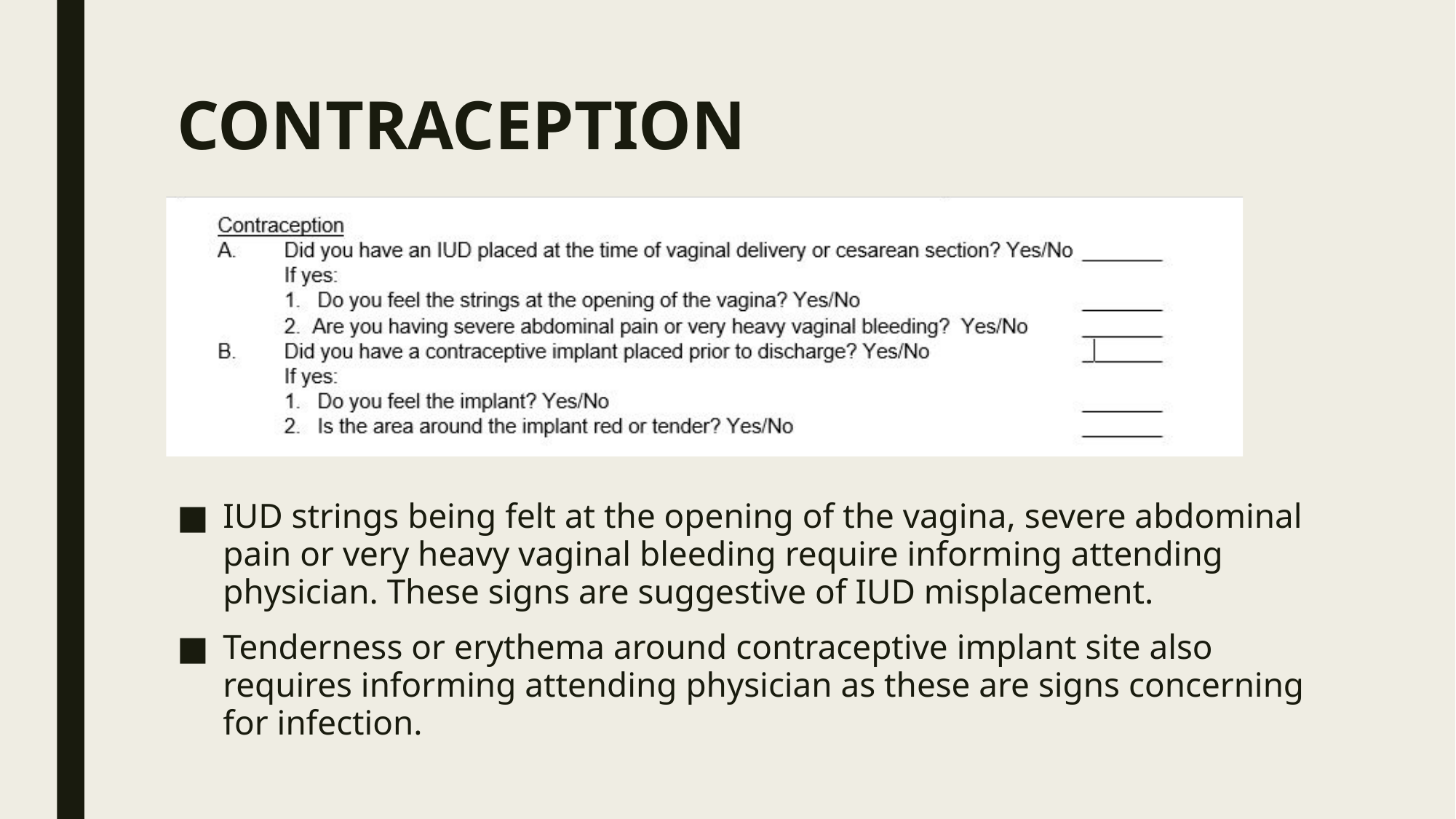

# CONTRACEPTION
IUD strings being felt at the opening of the vagina, severe abdominal pain or very heavy vaginal bleeding require informing attending physician. These signs are suggestive of IUD misplacement.
Tenderness or erythema around contraceptive implant site also requires informing attending physician as these are signs concerning for infection.

## Slide 11
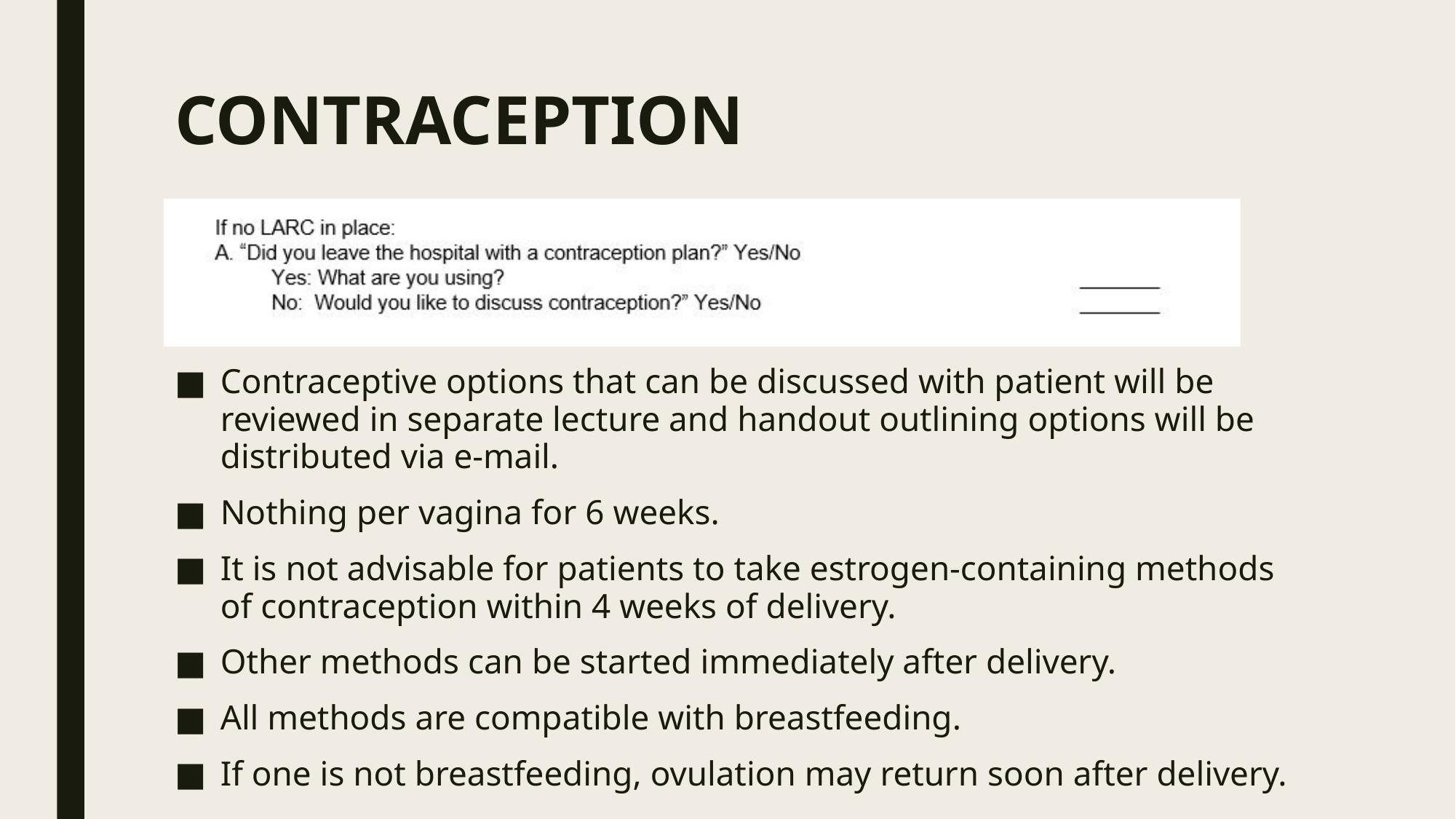

# CONTRACEPTION
Contraceptive options that can be discussed with patient will be reviewed in separate lecture and handout outlining options will be distributed via e-mail.
Nothing per vagina for 6 weeks.
It is not advisable for patients to take estrogen-containing methods of contraception within 4 weeks of delivery.
Other methods can be started immediately after delivery.
All methods are compatible with breastfeeding.
If one is not breastfeeding, ovulation may return soon after delivery.

## Slide 12
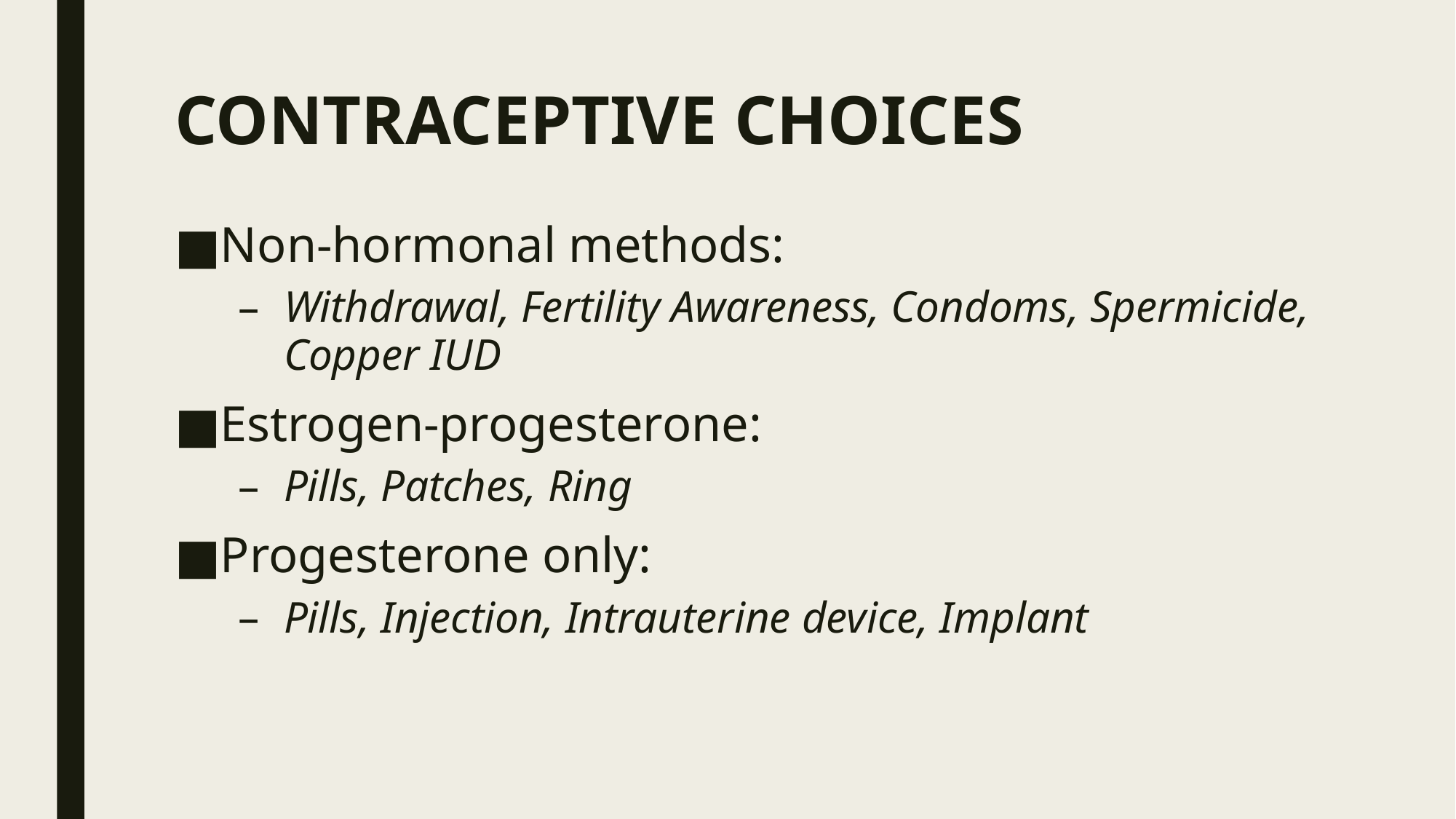

# CONTRACEPTIVE CHOICES
Non-hormonal methods:
Withdrawal, Fertility Awareness, Condoms, Spermicide, Copper IUD
Estrogen-progesterone:
Pills, Patches, Ring
Progesterone only:
Pills, Injection, Intrauterine device, Implant

## Slide 13
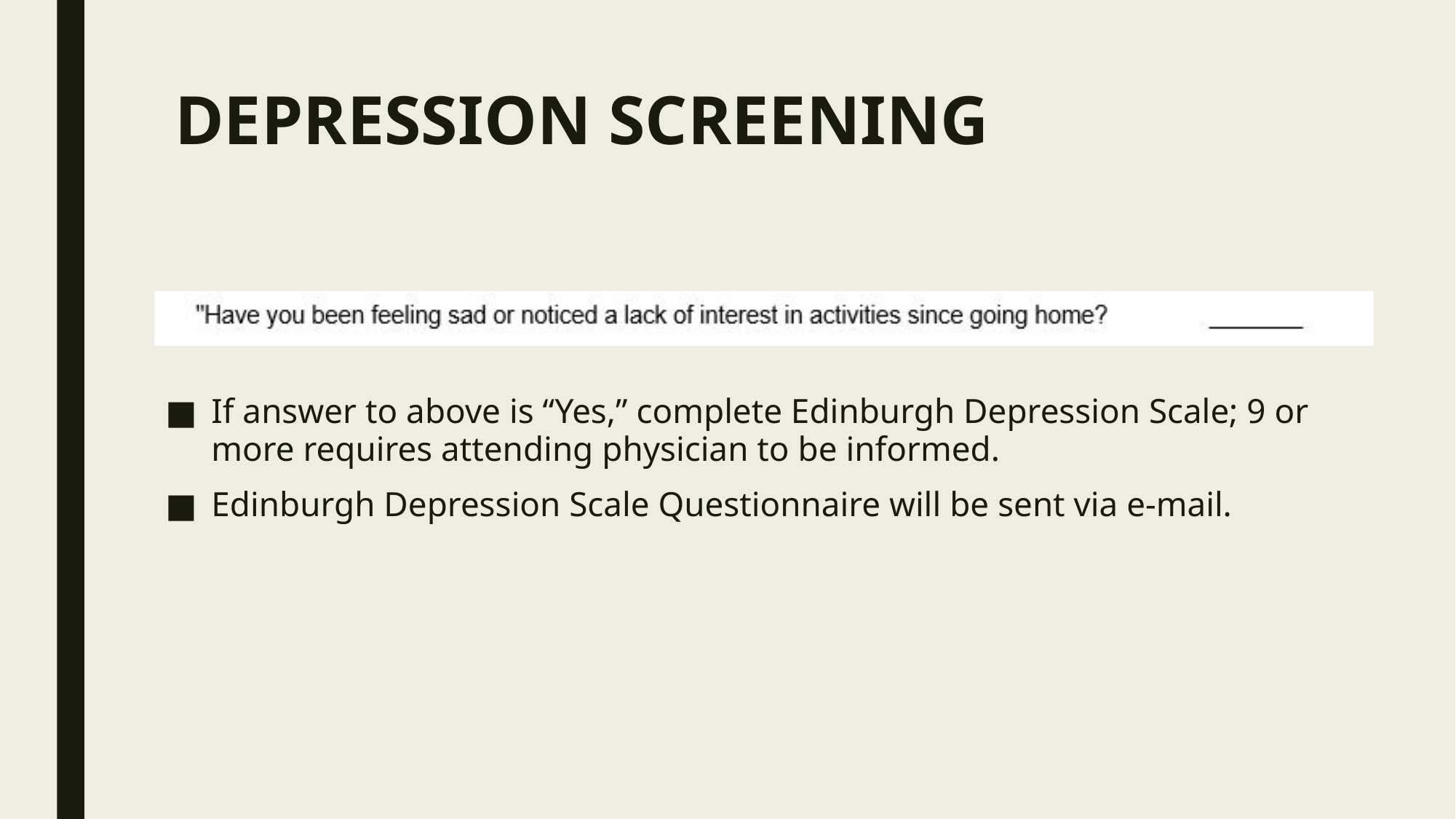

# DEPRESSION SCREENING
If answer to above is “Yes,” complete Edinburgh Depression Scale; 9 or more requires attending physician to be informed.
Edinburgh Depression Scale Questionnaire will be sent via e-mail.

## Slide 14
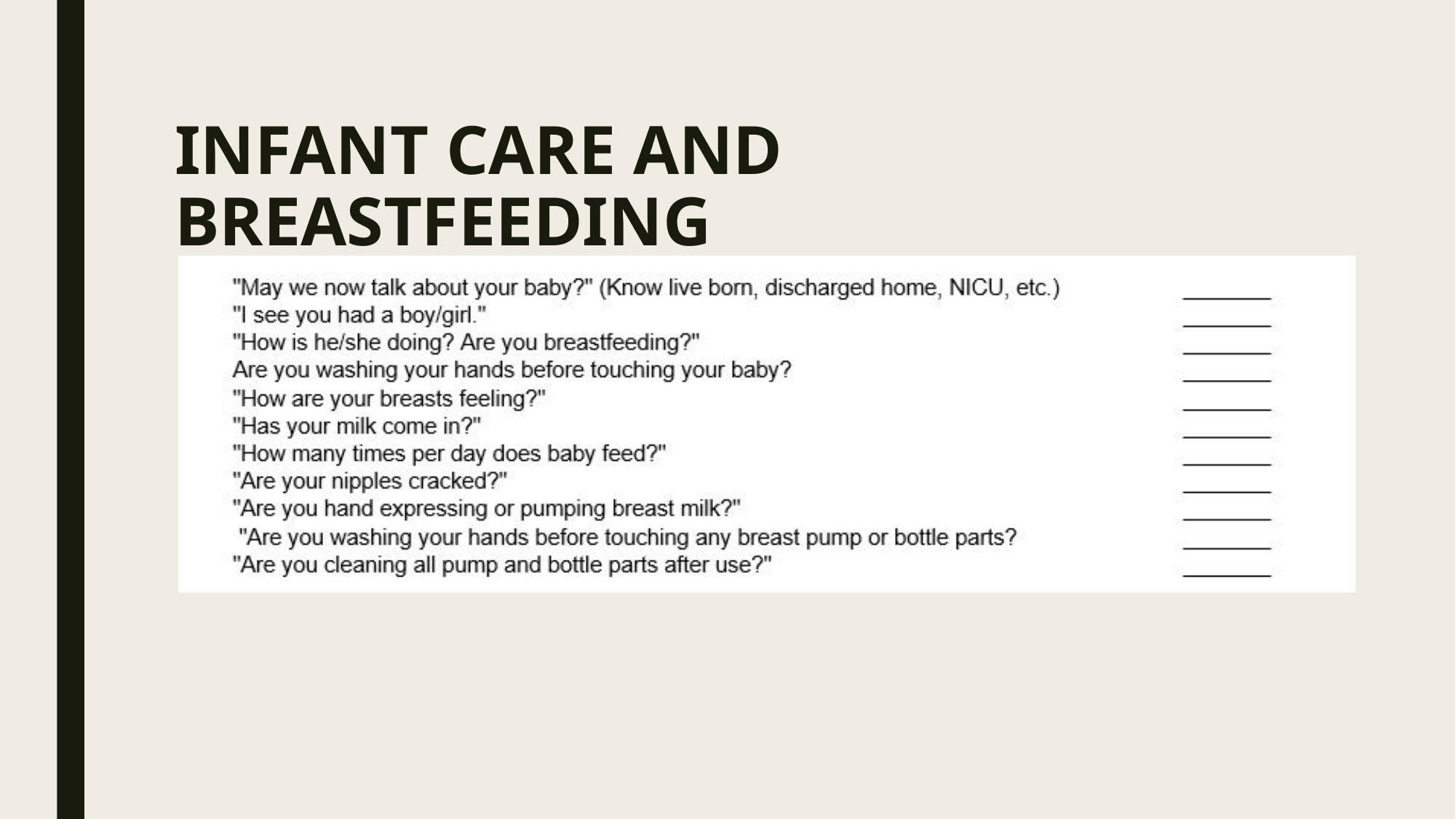

# INFANT CARE AND BREASTFEEDING

## Slide 15
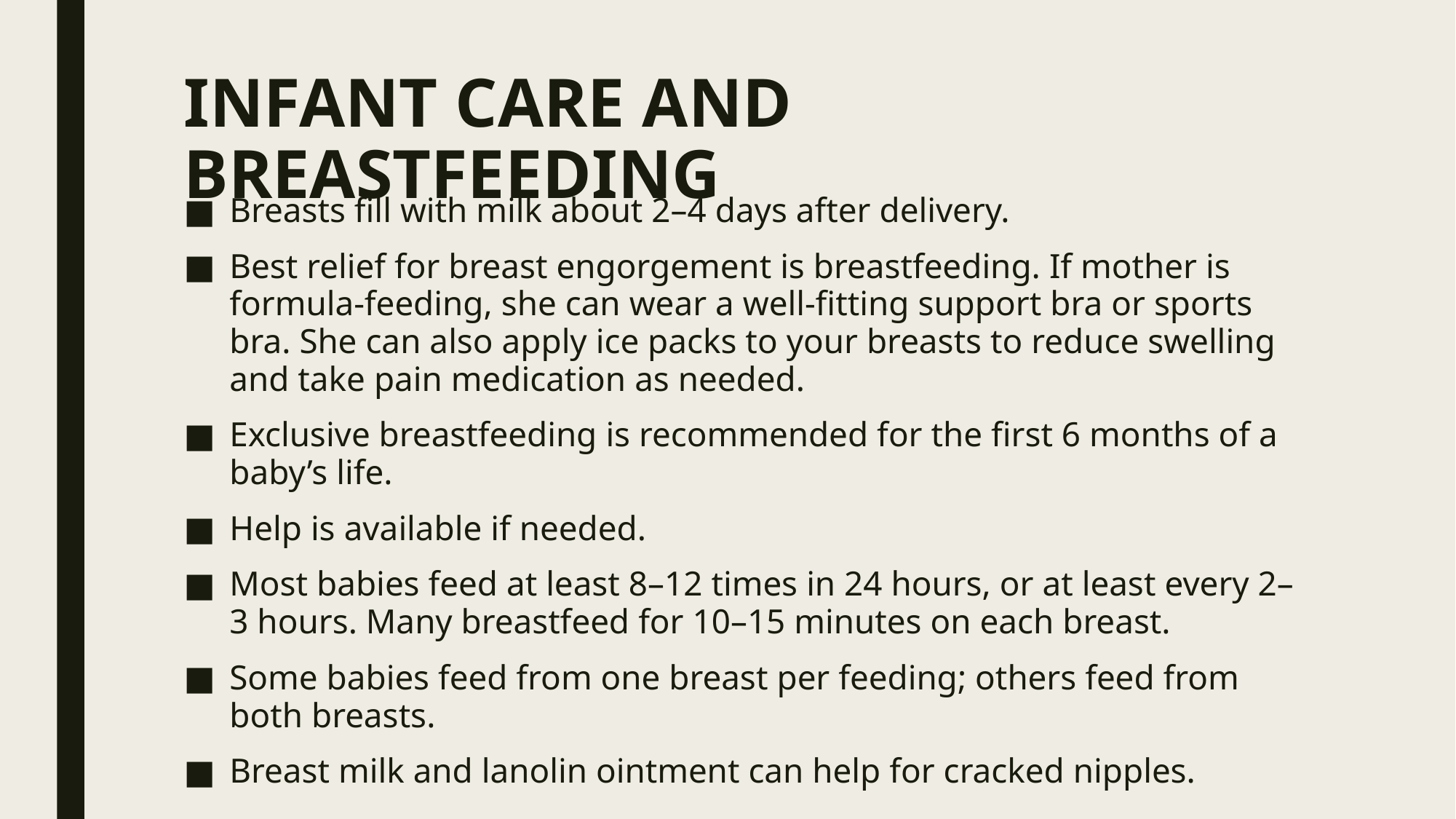

# INFANT CARE AND BREASTFEEDING
Breasts fill with milk about 2–4 days after delivery.
Best relief for breast engorgement is breastfeeding. If mother is formula-feeding, she can wear a well-fitting support bra or sports bra. She can also apply ice packs to your breasts to reduce swelling and take pain medication as needed.
Exclusive breastfeeding is recommended for the first 6 months of a baby’s life.
Help is available if needed.
Most babies feed at least 8–12 times in 24 hours, or at least every 2–3 hours. Many breastfeed for 10–15 minutes on each breast.
Some babies feed from one breast per feeding; others feed from both breasts.
Breast milk and lanolin ointment can help for cracked nipples.

## Slide 16
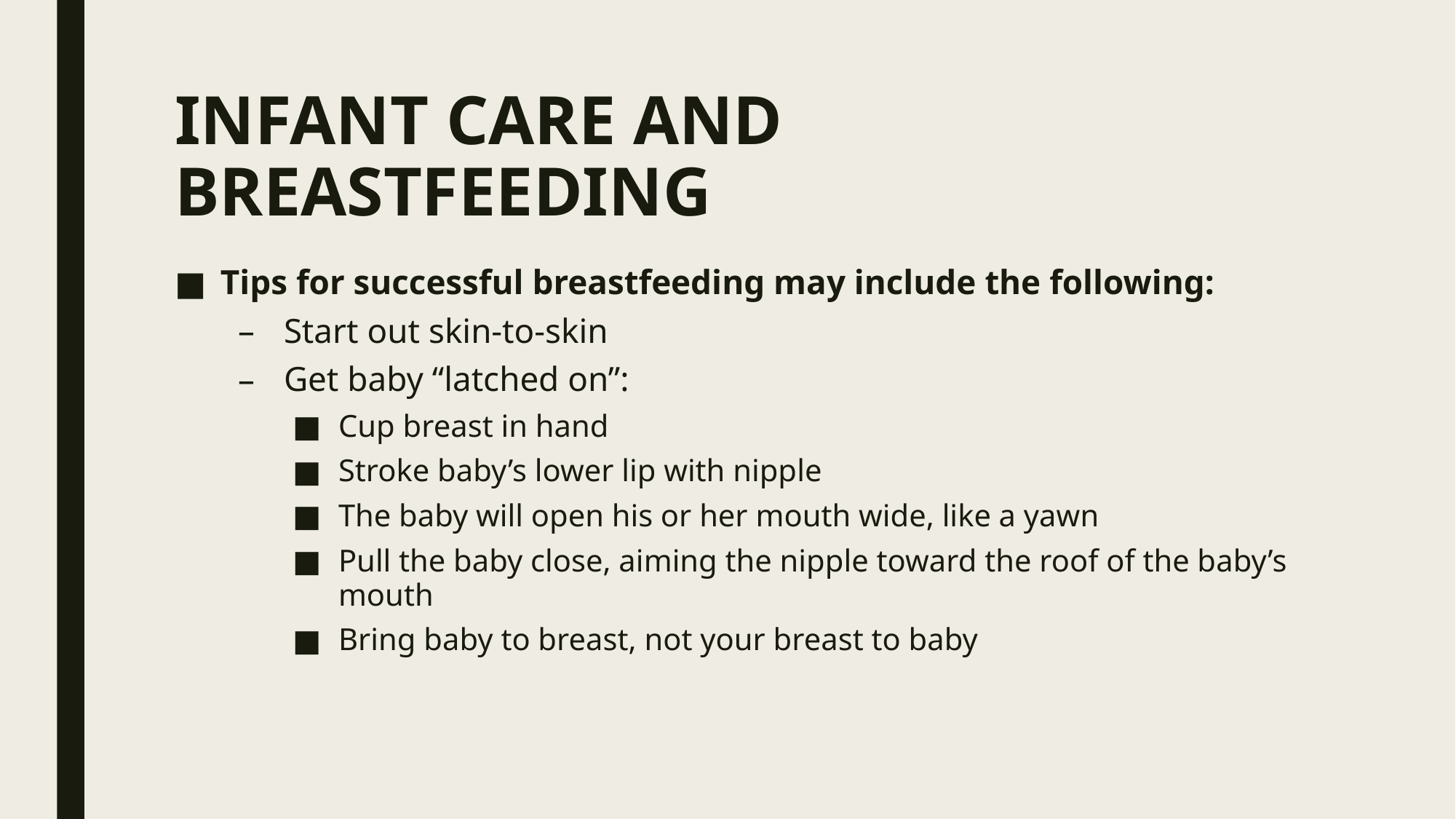

# INFANT CARE AND BREASTFEEDING
Tips for successful breastfeeding may include the following:
Start out skin-to-skin
Get baby “latched on”:
Cup breast in hand
Stroke baby’s lower lip with nipple
The baby will open his or her mouth wide, like a yawn
Pull the baby close, aiming the nipple toward the roof of the baby’s mouth
Bring baby to breast, not your breast to baby

## Slide 17
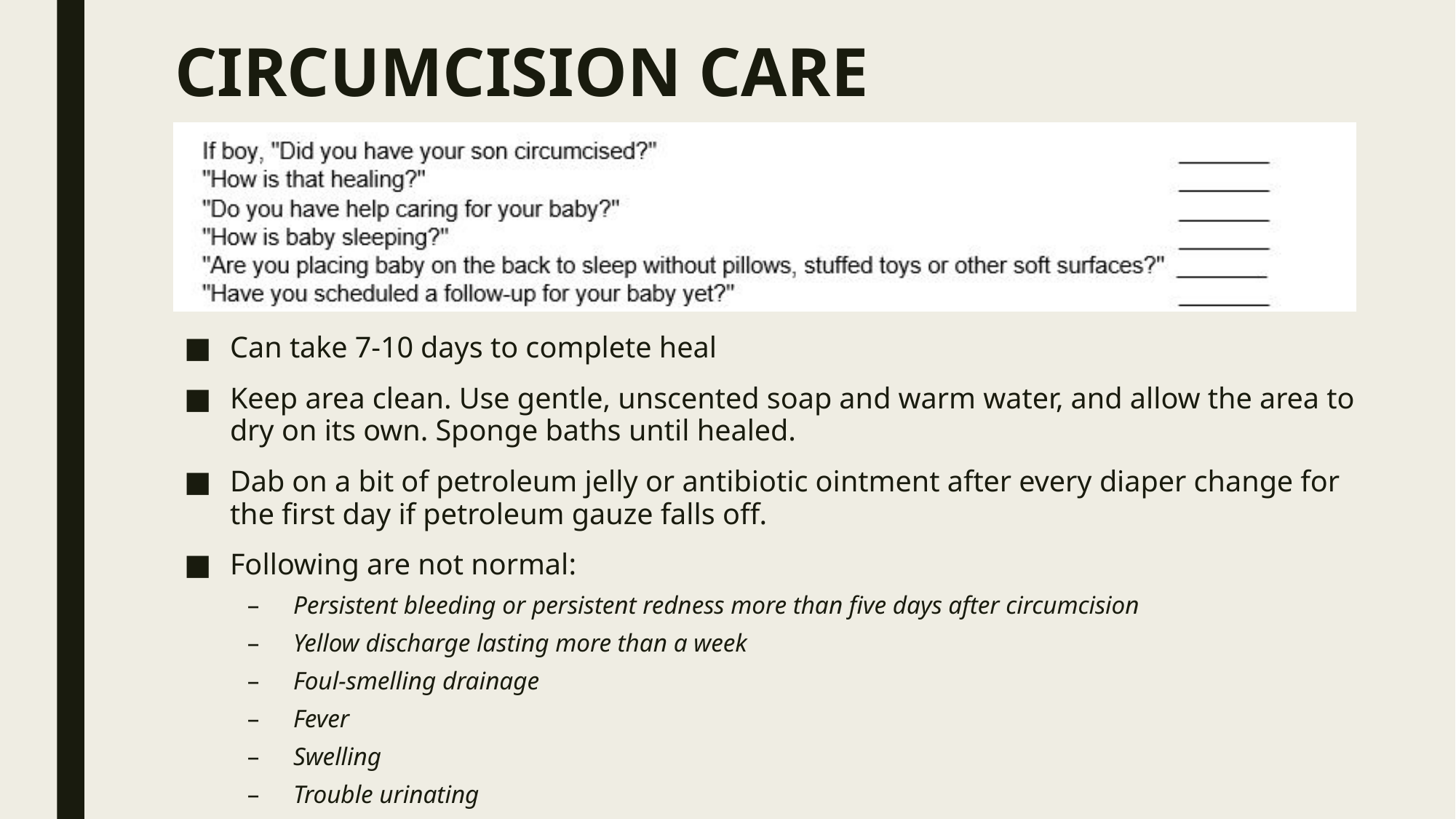

# CIRCUMCISION CARE
Can take 7-10 days to complete heal
Keep area clean. Use gentle, unscented soap and warm water, and allow the area to dry on its own. Sponge baths until healed.
Dab on a bit of petroleum jelly or antibiotic ointment after every diaper change for the first day if petroleum gauze falls off.
Following are not normal:
Persistent bleeding or persistent redness more than five days after circumcision
Yellow discharge lasting more than a week
Foul-smelling drainage
Fever
Swelling
Trouble urinating

## Slide 18
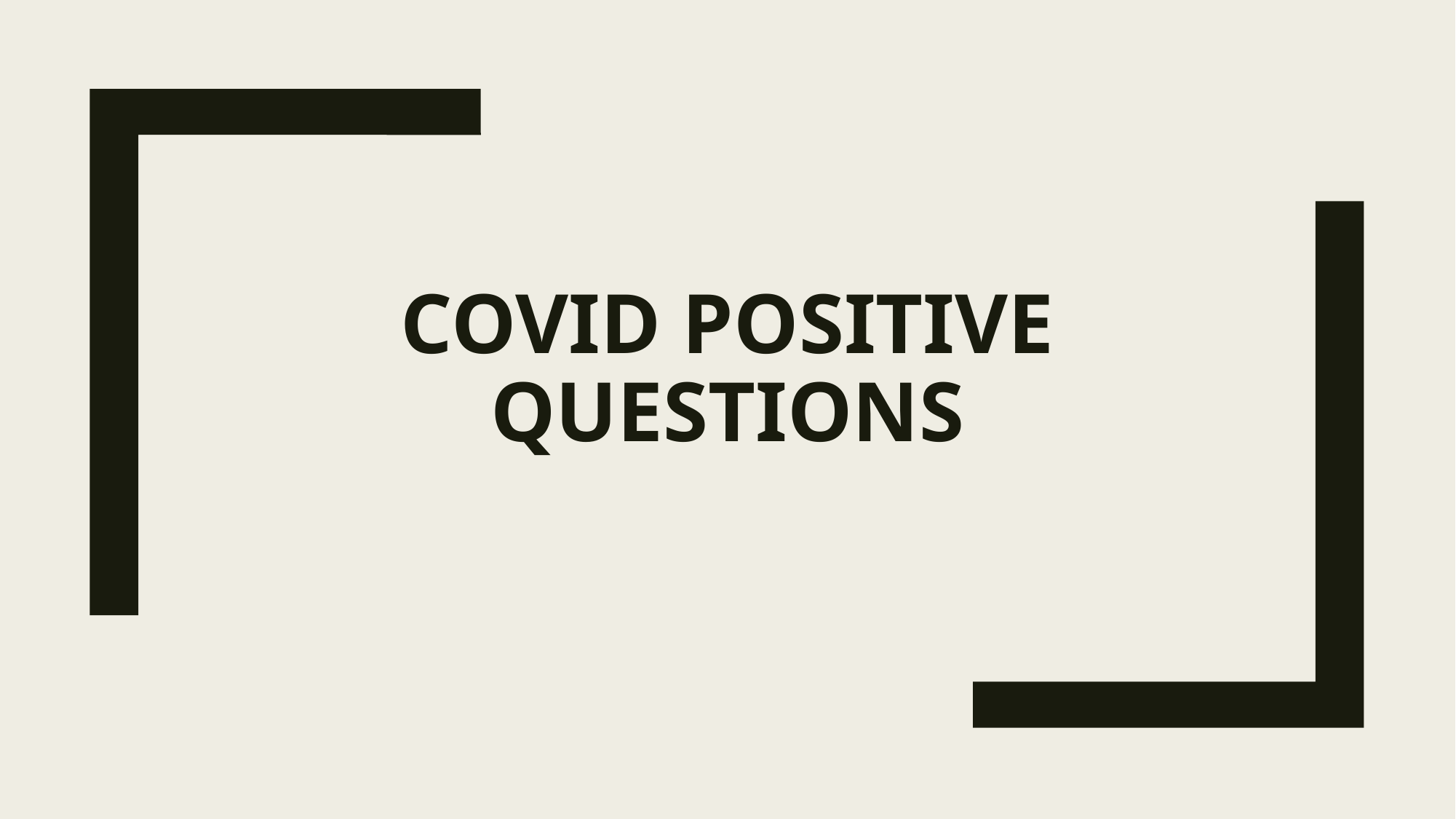

# COVID POSITIVE QUESTIONS

## Slide 19
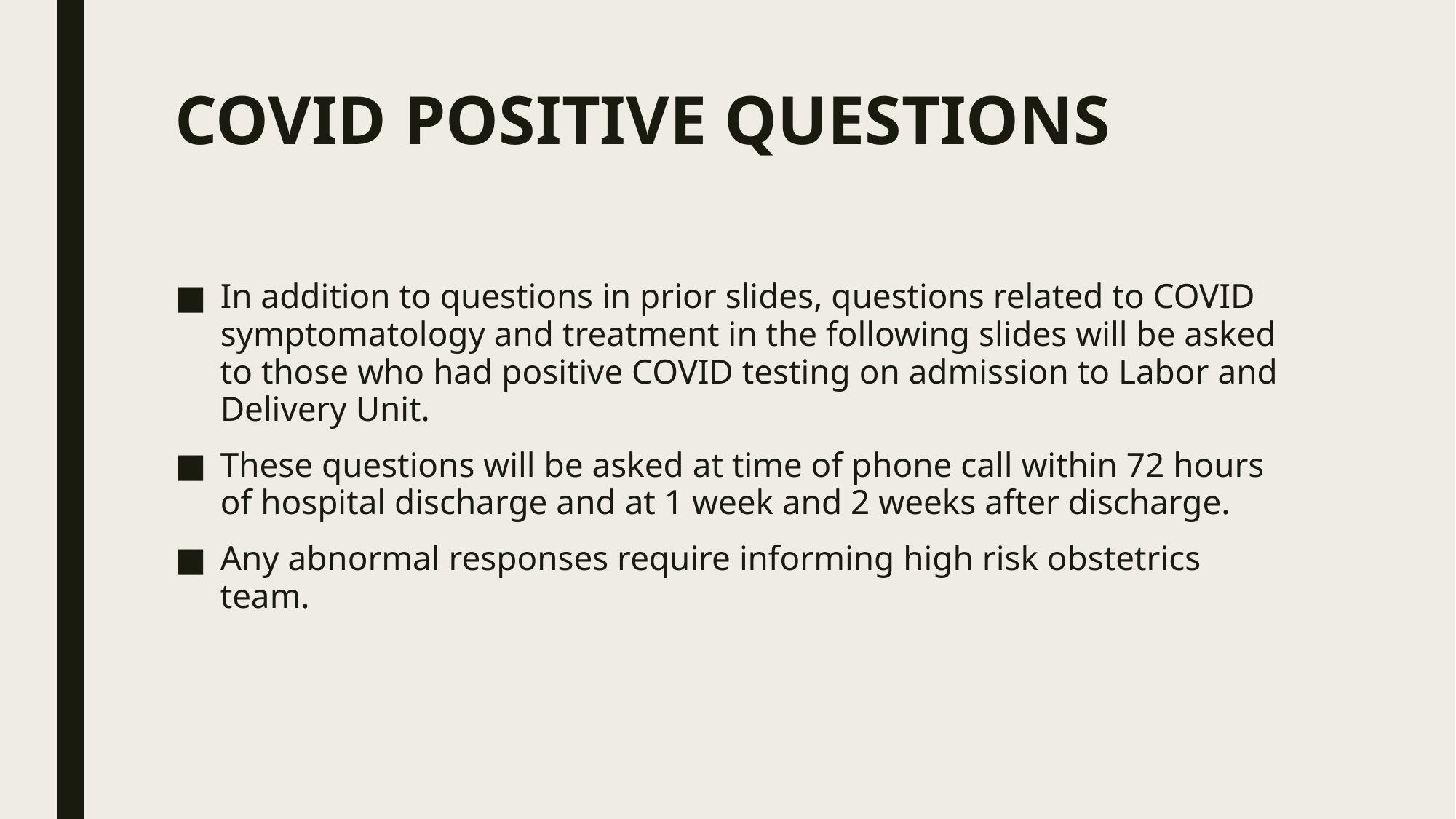

# COVID POSITIVE QUESTIONS
In addition to questions in prior slides, questions related to COVID symptomatology and treatment in the following slides will be asked to those who had positive COVID testing on admission to Labor and Delivery Unit.
These questions will be asked at time of phone call within 72 hours of hospital discharge and at 1 week and 2 weeks after discharge.
Any abnormal responses require informing high risk obstetrics team.

## Slide 20
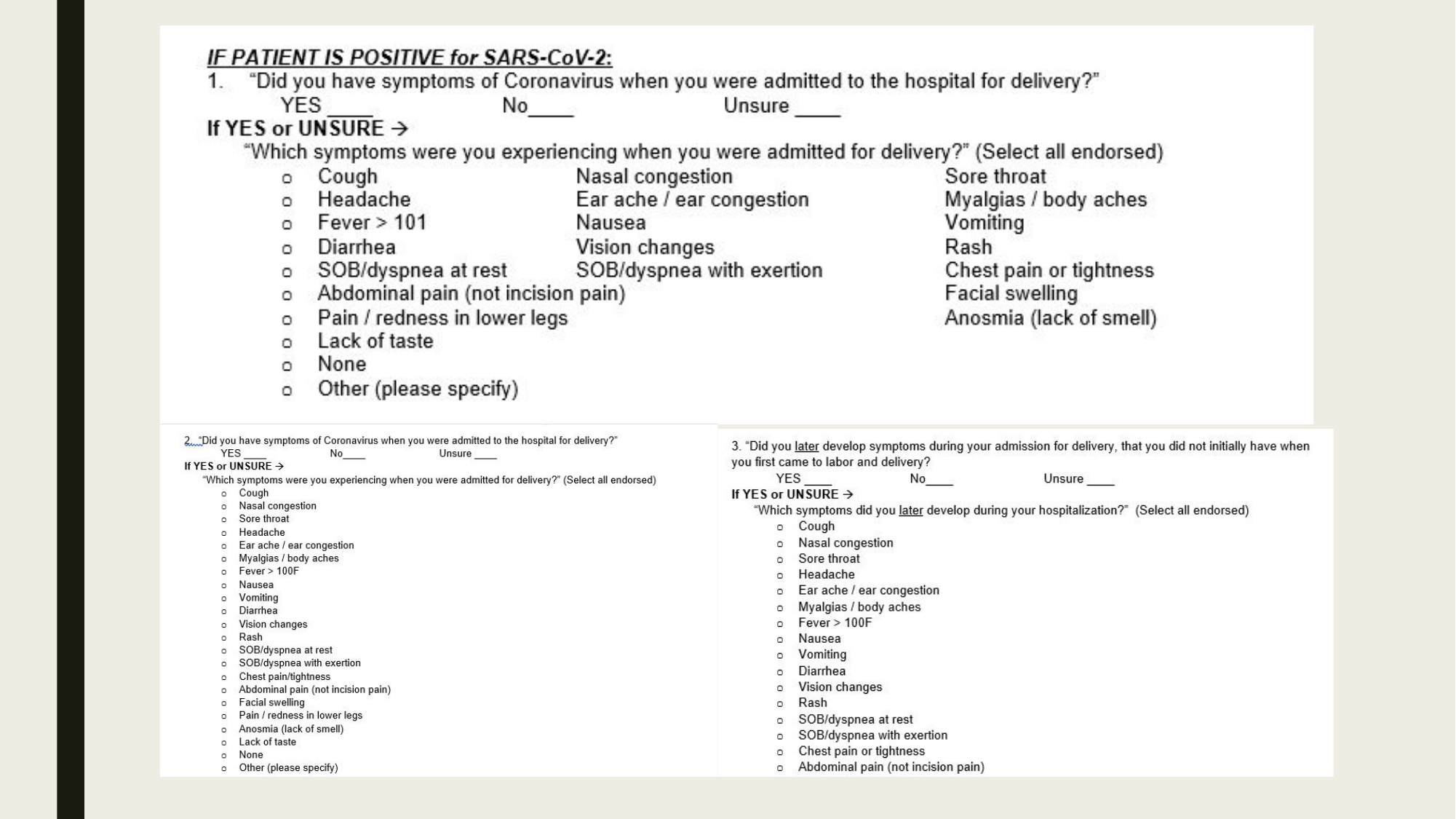

#

## Slide 21
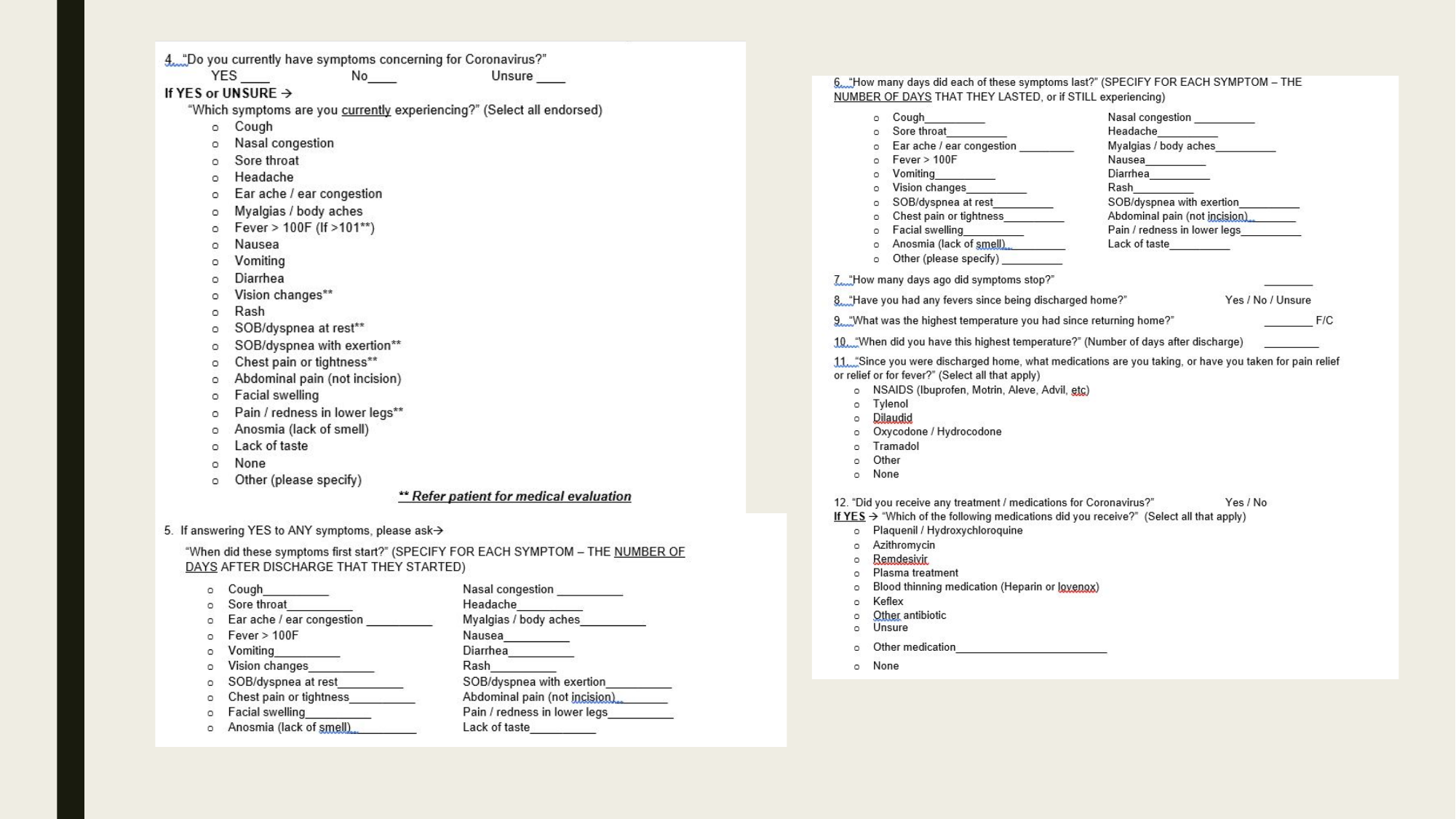

## Slide 22
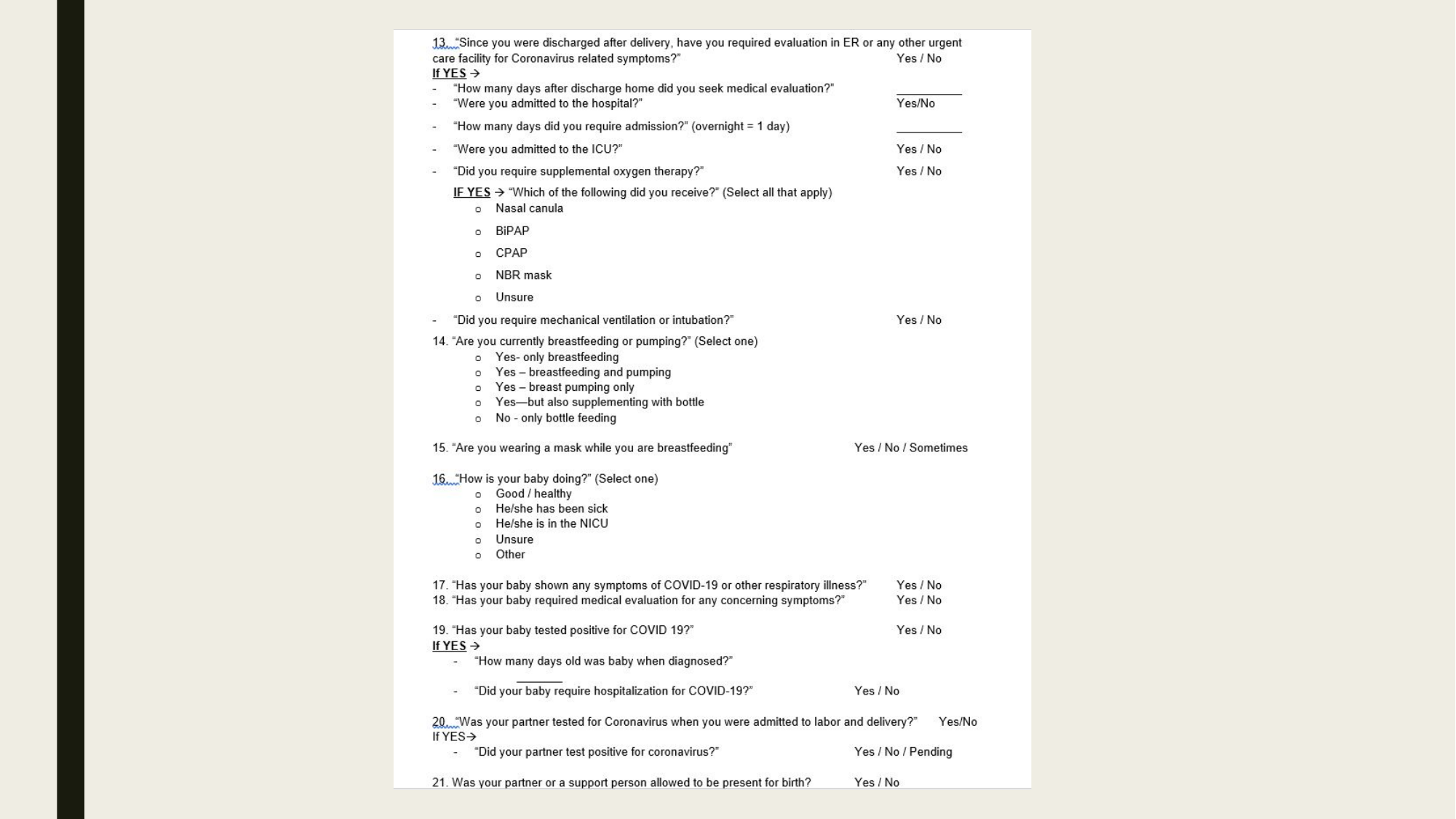

Supplement: Supplementary file 1 — Medical Student Postpartum Project.pptxCOVID Negative 72-Hour Follow-up.docxCOVID Positive 72-Hour Follow-up.docxAdditional Guidance.docxCOVID Positive 1- to 2-Week Follow-up.docx [file mep_2374-8265.11109-s001.zip › A. Medical Student Postpartum Project.pptx]
