# Supplementary material for: Successful Implementation of a Medical Student Postpartum Follow-up Phone Call Project
Source: MedEdPORTAL. 2021 Feb 19;17:11109. doi: 10.15766/mep_2374-8265.11109 (PMC7901253; doi:10.15766/mep_2374-8265.11109)
Supplement: Supplementary file 1 — Medical Student Postpartum Project.pptxCOVID Negative 72-Hour Follow-up.docxCOVID Positive 72-Hour Follow-up.docxAdditional Guidance.docxCOVID Positive 1- to 2-Week Follow-up.docx [file mep_2374-8265.11109-s001.zip › B. COVID Negative 72-Hour Follow-up.docx]

**Department of Obstetrics, Gynecology and Reproductive Sciences Post-Discharge Call Checklist**

“Hello, is this (Patient First Name, Patient Last Name)? (Confirm date of birth). This is ___________***calling*** from_____. I am a member of the health care team. May we talk for a few minutes about how you’re doing after your child's birth?”

“I see you had ***vaginal birth/cesarean birth***.” _____________

“Do you have any pain?” _______

“How would you describe your pain on a scale of 1 -10?” _______

***(If 7-8 out of 10 after taking pain medications, escalate)***

“Were you able to obtain all your medications?” _______

“Are prescribed medications working well?” _______

“How often do you need them?” _____________

*For cesarean delivery*:

“Please check your incision. Is it tender, is there any redness or warmth?” _______

“Do you have significant pain or leakage (drainage) from the incision site?” _______

“Has bleeding decreased?” _______

“How many pads are you using a day?” _______

***(Changing 1-2 pads every 1-2 hours is not normal, escalate)***

“Are you passing any clots?” _______

“If so, what size?“ ***(clots larger than golf ball are not normal)*** _______

“Any foul odors from the bleeding?” _______

***(If patient has fever – temperature 38C or higher, escalate)***

“Are you still using the peri-care bottle to keep that area clean?” _______

“Are you practicing social distancing at home?” _______

‘Please make sure that you continue to take precautions.”

*If patient had elevated blood pressures*,

“Have you checked your blood pressure since leaving the hospital?” _______

“If you were prescribed blood pressure medicine, are you taking it?” _______

***(If patient has any of the following with no improvement after taking medication, escalate)***

“Do you have a headache?” _______

“Blurry vision?” _______

“Increased swelling?” _______

“Chest pain or heartburn?” _______

“Do you have nausea and/or vomiting?” _______

“Are you following the instructions that were provided to you before discharge? _______

“How often are you urinating?” _______

“Any difficulty starting or stopping urinating?” _______

“Have you had a bowel movement?” _______

“How is your appetite?” _______

“Are you still taking your prenatal vitamins or any other medications prescribed after delivery?” _______

Contraception

A. Did you have an IUD placed at the time of vaginal delivery or cesarean section? Yes/No _______

If yes:

1. Do you feel the strings at the opening of the vagina? Yes/No _______

2. Are you having severe abdominal pain or very heavy vaginal bleeding? Yes/No _______

B. Did you have a contraceptive implant placed prior to discharge? Yes/No _______

If yes:

1. Do you feel the implant? Yes/No _______

2. Is the area around the implant red or tender? Yes/No _______

If no LARC in place:

A. “Did you leave the hospital with a contraception plan?” Yes/No

Yes: What are you using? _______

No: Would you like to discuss contraception?” Yes/No _______

“Have you been feeling sad or noticed a lack of interest in activities since going home? _______

***(If answer to above is “Yes,” complete Edinburgh Depression Scale, 9 or more requires text to point-person, positive answer to #10 requires escalation)***

“May we now talk about your baby?” (Know live born, discharged home, NICU, etc.) _______

“I see you had a ***boy/girl***.” _______

“How is he/she doing? Are you breastfeeding?” _______

Are you washing your hands before touching your baby? _______

“How are your breasts feeling?” _______

“Has your milk come in?” _______

“How many times per day does baby feed?” _______

“Are your nipples cracked?” _______

“Are you hand expressing or pumping breast milk?” _______

“Are you washing your hands before touching any breast pump or bottle parts? _______

“Are you cleaning all pump and bottle parts after use?” _______

If boy, “Did you have your son circumcised?” _______

“How is that healing?” _______

“Do you have help caring for your baby?” _______

“How is baby sleeping?” _______

“Are you placing baby on the back to sleep without pillows, stuffed toys or other soft surfaces?” _______

“Have you scheduled a follow-up for your baby yet?” _______

“I know I’ve asked many questions, and I am glad to hear you are doing well (if this is accurate).”

“Do you have any questions for me?” “These are trying times and we are here for you. Stay safe.”

“If your partner is positive for coronavirus, I recommend he/she/they continue to take precautions and seek medical guidance from his/her/them health care provider.”
