# Supplementary material for: Successful Implementation of a Medical Student Postpartum Follow-up Phone Call Project
Source: MedEdPORTAL. 2021 Feb 19;17:11109. doi: 10.15766/mep_2374-8265.11109 (PMC7901253; doi:10.15766/mep_2374-8265.11109)
Supplement: Supplementary file 1 — Medical Student Postpartum Project.pptxCOVID Negative 72-Hour Follow-up.docxCOVID Positive 72-Hour Follow-up.docxAdditional Guidance.docxCOVID Positive 1- to 2-Week Follow-up.docx [file mep_2374-8265.11109-s001.zip › C. COVID Positive 72-Hour Follow-up.docx]

**Department of Obstetrics, Gynecology and Reproductive Sciences Post-Discharge Call Checklist**

**Date of interview:** / / /

**Postpartum:** 48-72 hrs _____

“Hello, is this (Patient First Name, Patient Last Name)? (Confirm date of birth). This is ___________***calling*** from ***_______***. I am a member of the health care team. May we talk for a few minutes about how you’re doing after your child's birth?”

“I see you had ***vaginal birth/cesarean birth***.”

“Do you have any pain?” Yes / No

“How would you describe your pain on a scale of 1 -10?” _______

***(If 7-8 out of 10 after taking pain medications, escalate)***

“Were you able to obtain all your medications?” Yes / No

“Which medications are you currently taking?”

____________________________________________________________________________

“Are prescribed medications helping with your pain?” Yes / No

“How often do you need them?” _______

***For cesarean delivery*:**

“Please check your incision. Is it tender, is there any redness or warmth?” _______

“Do you have significant pain or leakage (drainage) from the incision site?” _______

“Has bleeding decreased?” _______

“How many pads are you using a day?” _______

***(Changing 1-2 pads every 1-2 hours is not normal, escalate)***

“Are you passing any clots?” _______

“If so, what size?“ ***(clots larger than golf ball are not normal)*** _______

“Any foul odors from the bleeding?” _______

***(If patient has fever – temperature 38C or higher, escalate)***

“Are you still using the peri-care bottle to keep that area clean?” _______

***IF PATIENT IS POSITIVE for SARS-CoV-2*:**

1. “Did you have symptoms of Coronavirus when you were admitted to the hospital for delivery?”

YES ____ No____ Unsure ____

**If YES or UNSURE** 🡪

“Which symptoms were you experiencing when you were admitted for delivery?” (Select all endorsed)

- - Cough Nasal congestion Sore throat
  - Headache Ear ache / ear congestion Myalgias / body aches
  - Fever > 101 Nausea Vomiting
  - Diarrhea Vision changes Rash
  - SOB/dyspnea at rest SOB/dyspnea with exertion Chest pain or tightness
  - Abdominal pain (not incision pain) Facial swelling
  - Pain / redness in lower legs Anosmia (lack of smell)
  - Lack of taste
  - None
  - Other (please specify)

1. “Did you later develop symptoms during your admission for delivery, that you did not initially have when you first came to labor and delivery?

**If YES or UNSURE** 🡪

“Which symptoms did you later develop during your hospitalization?” (Select all endorsed)

- - Cough Nasal congestion Sore throat
  - Headache Ear ache / ear congestion Myalgias / body aches
  - Fever > 101 Nausea Vomiting
  - Diarrhea Vision changes Rash
  - SOB/dyspnea at rest SOB/dyspnea with exertion Chest pain or tightness
  - Abdominal pain (not incision pain) Facial swelling
  - Pain / redness in lower legs Anosmia (lack of smell)
  - Lack of taste
  - None
  - Other (please specify)

1. “Do you currently have symptoms concerning for Coronavirus?”

YES ____ No____ Unsure ____

**If YES or UNSURE** 🡪

“Which symptoms are you currently experiencing?” (Select all endorsed)

- - Cough Nasal congestion Sore throat
  - Headache Ear ache / ear congestion Myalgias / body aches
  - Fever > 101** Nausea Vomiting
  - Diarrhea Vision changes** Rash
  - SOB/dyspnea at rest** SOB/dyspnea with exertion** Chest pain or tightness**
  - Abdominal pain (not incision) Facial swelling
  - Pain / redness in lower legs** Anosmia (lack of smell)
  - Lack of taste None
  - Other (please specify)

***** Refer patient for medical evaluation***

1. If answering YES to ANY symptoms, please ask🡪

“When did these symptoms first start?” (SPECIFY FOR EACH SYMPTOM – THE NUMBER OF DAYS AFTER DISCHARGE THAT THEY STARTED)

- - Cough_____­­­_____ Nasal congestion _____­­­_____
  - Sore throat_____­­­_____ Headache_____­­­_____
  - Ear ache / ear congestion _____­­­_____ Myalgias / body aches_____­­­_____
  - Fever > 101 Nausea_____­­­_____
  - Vomiting_____­­­_____ Diarrhea_____­­­_____
  - Vision changes_____­­­_____ Rash_____­­­_____
  - SOB/dyspnea at rest_____­­­_____ SOB/dyspnea with exertion_____­­­_____
  - Chest pain or tightness_____­­­_____ Abdominal pain (not incision)_____­­­___
  - Facial swelling_____­­­_____ Pain / redness in lower legs_____­­­_____
  - Anosmia (lack of smell)_____­­­_____ Lack of taste_____­­­_____
  - Other (please specify) _____­­­_____

1. “How many days did each of these symptoms last?” (SPECIFY FOR EACH SYMPTOM – THE NUMBER OF DAYS THAT THEY LASTED, or if STILL experiencing)
   - Cough_____­­­_____ Nasal congestion _____­­­_____
   - Sore throat_____­­­_____ Headache_____­­­_____
   - Ear ache / ear congestion _____­­­_____ Myalgias / body aches_____­­­_____
   - Fever > 101 Nausea_____­­­_____
   - Vomiting_____­­­_____ Diarrhea_____­­­_____
   - Vision changes_____­­­_____ Rash_____­­­_____
   - SOB/dyspnea at rest_____­­­_____ SOB/dyspnea with exertion_____­­­_____
   - Chest pain or tightness_____­­­_____ Abdominal pain (not incision) _____­­­___
   - Facial swelling_____­­­_____ Pain / redness in lower legs_____­­­_____
   - Anosmia (lack of smell)_____­­­_____ Lack of taste_____­­­_____
   - Other (please specify) _____­­­_____
2. “How many days ago did symptoms stop?” ________
3. “Have you had any fevers since being discharged home?” Yes / No / Unsure
4. “What was the highest temperature you had since returning home?” ________ F/C
5. “When did you have this highest temperature?” (Number of days after discharge) _________
6. “Since you were discharged home, what medications are you taking, or have you taken for pain relief or relief or for fever?” (Select all that apply)

- NSAIDS (Ibuprofen, Motrin, Aleve, Advil, etc)
- Tylenol
- Dilaudid
- Oxycodone / Hydrocodone
- Tramadol
- Other
- None

1. “Did you receive any treatment / medications for Coronavirus?” Yes / No

**If YES** 🡪 “Which of the following medications did you receive?” (Select all that apply)

- Plaquenil / Hydroxychloroquine
- Azithromycin
- Remdesivir
- Plasma treatment
- Blood thinning medication (Heparin or lovenox)
- Keflex
- Other antibiotic
- Unsure
- Other medication
- None

1. “Since you were discharged after delivery, have you required evaluation in ER or any other urgent care facility for Coronavirus related symptoms?”

Yes / No

**If YES** 🡪

- “How many days after discharge home did you seek medical evaluation?” __________
- “Were you admitted to the hospital?” Yes/No
- “How many days did you require admission?” (overnight = 1 day) __________
- “Were you admitted to the ICU?” Yes / No
- “Did you require supplemental oxygen therapy?” Yes / No

**IF YES** 🡪 “Which of the following did you receive?” (Select all that apply)

- Nasal canula
- BiPAP
- CPAP
- NBR mask
- Unsure
- “Did you require mechanical ventilation or intubation?” Yes / No

1. “Are you currently breastfeeding or pumping?” (Select one)
   - Yes- only breastfeeding
   - Yes – breastfeeding and pumping
   - Yes – breast pumping only
   - Yes—but also supplementing with bottle
   - No - only bottle feeding
2. “Are you wearing a mask while you are breastfeeding” Yes / No / Sometimes
3. “How is your baby doing?” (Select one)
   - Good / healthy
   - He/she has been sick
   - He/she is in the NICU
   - Unsure
   - Other
4. “Has your baby shown any symptoms of COVID-19 or other respiratory illness?” Yes / No
5. “Has your baby required medical evaluation for any concerning symptoms?” Yes / No
6. “Has your baby tested positive for COVID 19?” Yes / No

**If YES** 🡪

- “How many days old was baby when diagnosed?” _______
- “Did your baby require hospitalization for COVID-19?” Yes / No

1. “Since delivery, have you experienced any of the following symptoms?” (Select all that apply)
   - New onset or worsening anxiety**
   - New onset or worsening depression**
   - Difficulty sleeping (aside from waking to care for baby)
   - None

***** Refer patient to resources***

1. “Are you wearing a mask around others?” Yes / No / Sometimes
2. “Are you practicing social distancing at home?” Yes / No / Sometimes
3. Are you washing your hands before touching your baby?
   - Yes with soap and water
   - Yes with hand sanitizer
   - Yes, with soap and water and hand sanitizer
   - Sometimes
   - No

‘Please make sure that you continue to take precautions.”

***END OF SARS-CoV-2* *SECTION***

*If patient had elevated blood pressures*,

“Have you checked your blood pressure since leaving the hospital?” _______

“If you were prescribed blood pressure medicine, are you taking it?” _______

***(If patient has any of the following with no improvement after taking medication, escalate)***

“Do you have a headache?” _______

“Blurry vision?” _______

“Increased swelling?” _______

“Chest pain or heartburn?” _______

“Do you have nausea and/or vomiting?” _______

“Are you following the instructions that were provided to you before discharge? _______

“How often are you urinating?” _______

“Any difficulty starting or stopping urinating?” _______

“Have you had a bowel movement?” _______

“How is your appetite?” _______

“Are you still taking your prenatal vitamins or any other medications prescribed after delivery?” _______

Contraception

A. Did you have an IUD placed at the time of vaginal delivery or cesarean section? Yes/No _______

If yes:

1. Do you feel the strings at the opening of the vagina? Yes/No _______

2. Are you having severe abdominal pain or very heavy vaginal bleeding? Yes/No _______

B. Did you have a contraceptive implant placed prior to discharge? Yes/No _______

If yes:

1. Do you feel the implant? Yes/No _______

2. Is the area around the implant red or tender? Yes/No _______

If no LARC in place:

A. “Did you leave the hospital with a contraception plan?” Yes/No

Yes: What are you using? _______

No: Would you like to discuss contraception?” Yes/No _______

(If answer to above is “Yes,” review contraceptive options)

“Have you been feeling sad or noticed a lack of interest in activities since going home? _______

***(If answer to above is “Yes,” complete Edinburgh Depression Scale, 9 or more requires text to point-person, positive answer to #10 requires escalation)***

“May we now talk about your baby?” (Know live born, discharged home, NICU, etc.) _______

“I see you had a ***boy/girl***.” _______

“How is he/she doing?

“How are your breasts feeling?” _______

“How many times per day does baby feed?” _______

“Are your nipples cracked?” _______

If boy, “Did you have your son circumcised?” _______

“How is that healing?” _______

“Do you have help caring for your baby?” _______

“How is baby sleeping?” _______

“Are you placing baby on the back to sleep without pillows, stuffed toys or other soft surfaces?” _______

“Have you scheduled a follow-up for your baby yet?” _______

“I know I’ve asked many questions, and I am glad to hear you are doing well (if this is accurate).”

“Do you have any questions for me?” “These are trying times and we are here for you. Stay safe.”
