# Supplementary material for: Successful Implementation of a Medical Student Postpartum Follow-up Phone Call Project
Source: MedEdPORTAL. 2021 Feb 19;17:11109. doi: 10.15766/mep_2374-8265.11109 (PMC7901253; doi:10.15766/mep_2374-8265.11109)
Supplement: Supplementary file 1 — Medical Student Postpartum Project.pptxCOVID Negative 72-Hour Follow-up.docxCOVID Positive 72-Hour Follow-up.docxAdditional Guidance.docxCOVID Positive 1- to 2-Week Follow-up.docx [file mep_2374-8265.11109-s001.zip › D. Additional Guidance.docx]

**ADDITIONAL GUIDANCE WHEN CALLING PATIENTS**

**Perineal Care**

To help with perineal pain/swelling, patients can apply cold packs or chilled witch-hazel pads to the area. If sitting is uncomfortable, patients can sit on a pillow. Patients can also do Sitz baths (sitting in warm water that’s just deep enough to cover buttocks and hips. Special basins that can be filled with clean, warm water from the faucet and then placed on a toilet seat are made for this purpose.

**Bleeding**

Bleeding may last up to 4-6 weeks after delivery. For the first few days after delivery, bleeding is heavy and bright red. It may have a few small clots. A week after birth, bleeding often is pink or brown. Period may return 6-8 weeks after delivery or later if patient is breastfeeding.

**Difficulty Urinating**

May be due to swelling or pain. Recommend Sitz bath. When on the toilet, spraying warm water over genitals may can help trigger the flow of urine. Running the tap while in the bathroom may also help too. Patients should also be sure to drink plenty of fluids as well. This pain usually goes away within days of delivery.

**Urinary incontinence**

Very common. Recommend Kegel exercises. Patients may feel more comfortable wearing a sanitary pad until the problem goes away.

*Kegel Exercises*

Contract pelvic floor muscles for 3 to 5 seconds.

Relax for 3 to 5 seconds.

Repeat the contract/relax cycle 10 times.

Extend the contraction time. Gradually increase to 10-second contractions and relaxations.

Try to do at least 30 to 40 Kegel exercises every day. Spreading them throughout the day is better than doing them all at once.

**Constipation and painful gas**

Advise walking, eating food high in fiber and drinking plenty of fluids. Also recommend stool softeners. Colace which is over the counter can be taken twice a day as needed for constipation.

**Hemorrhoids**

May get worse after delivery. Medicated sprays or ointments, sitz baths, and cold witch-hazel compresses can help.

**Contraception**

Nothing per vagina for 6 weeks. It is not advisable for patients to take estrogen-containing methods of contraception within 4 weeks of delivery. Other methods can be started immediately after delivery. All methods are compatible with breastfeeding. If one is not breastfeeding, ovulation may return soon after delivery.

**Breastfeeding**

Breasts fill with milk about 2–4 days after delivery. Best relief for breast engorgement is breastfeeding. If mother is formula-feeding, she can wear a well-fitting support bra or sports bra. She can also apply ice packs to your breasts to reduce swelling and take pain medication as needed.

Exclusive breastfeeding is recommended for the first 6 months of a baby’s life.

Help is available if needed.

Tips for successful breastfeeding may include the following:

• Start out skin-to-skin

• Get baby “latched on”: Cup breast in hand, stroke baby’s lower lip with nipple. The baby will open his or her mouth wide, like a yawn. Pull the baby close, aiming the nipple toward the roof of the baby’s mouth. Bring baby to breast, not your breast to baby.

Most babies feed at least 8–12 times in 24 hours, or at least every 2–3 hours. Many breastfeed for 10–15 minutes on each breast. Some babies feed from one breast per feeding; others feed from both breasts.

Breast milk and lanolin ointment can help for cracked nipples.

**Circumcision**

Can take 7-10 days to complete heal.

Keep area clean. Use gentle, unscented soap and warm water, and allow the area to dry on its own. Sponge baths until healed. Dab on a bit of petroleum jelly or antibiotic ointment after every diaper change for the first day if petroleum gauze falls off. Following are not normal: persistent bleeding or persistent redness more than five days after circumcision, yellow discharge lasting more than a week, foul-smelling drainage, fever, swelling, trouble urinating
