# Supplementary material for: Successful Implementation of a Medical Student Postpartum Follow-up Phone Call Project
Source: MedEdPORTAL. 2021 Feb 19;17:11109. doi: 10.15766/mep_2374-8265.11109 (PMC7901253; doi:10.15766/mep_2374-8265.11109)
Supplement: Supplementary file 1 — Medical Student Postpartum Project.pptxCOVID Negative 72-Hour Follow-up.docxCOVID Positive 72-Hour Follow-up.docxAdditional Guidance.docxCOVID Positive 1- to 2-Week Follow-up.docx [file mep_2374-8265.11109-s001.zip › E. COVID Positive 1- to 2-Week Follow-up.docx]

**Department of Obstetrics, Gynecology, and Reproductive Sciences**

**COVID Positive**

**Post-Discharge Call Checklist**

**Date of interview:**

**Patient MRN:**

**Postpartum/Discharge:** 1 week _____ 2 weeks _____

**Interviewer Name:**

“I see you had ***vaginal birth/cesarean birth***.”

“Do you have any pain?” Yes / No

“How would you describe your pain on a scale of 1 -10?” _______

***(If 7-8 out of 10 after taking pain medications, escalate)***

“Were you able to obtain all your medications?” Yes / No

“Which medications are you currently taking?”

____________________________________________________________________________

“Are prescribed medications working well?” Yes / No

“How often do you need them?”

____________________________________________________________________________

1. “Did you have symptoms of Coronavirus BEFORE you were admitted to the hospital for delivery?”

YES ____ No____ Unsure ____

**If YES or UNSURE** 🡪

“Which symptoms were you experiencing prior to admission for delivery?” (Select all endorsed)

- - Cough
  - Nasal congestion
  - Sore throat
  - Headache
  - Ear ache / ear congestion
  - Myalgias / body aches
  - Fever > 100F
  - Nausea
  - Vomiting
  - Diarrhea
  - Vision changes
  - Rash
  - SOB/dyspnea at rest
  - SOB/dyspnea with exertion
  - Chest pain or tightness
  - Abdominal pain (not incision pain)
  - Facial swelling
  - Pain / redness in lower legs
  - Anosmia (lack of smell)
  - Lack of taste
  - None
  - Other (please specify)

2. “Did you have symptoms of Coronavirus when you were admitted to the hospital for delivery?”

YES ____ No____ Unsure ____

**If YES or UNSURE** 🡪

“Which symptoms were you experiencing when you were admitted for delivery?” (Select all endorsed)

- - Cough
  - Nasal congestion
  - Sore throat
  - Headache
  - Ear ache / ear congestion
  - Myalgias / body aches
  - Fever > 100F
  - Nausea
  - Vomiting
  - Diarrhea
  - Vision changes
  - Rash
  - SOB/dyspnea at rest
  - SOB/dyspnea with exertion
  - Chest pain/tightness
  - Abdominal pain (not incision pain)
  - Facial swelling
  - Pain / redness in lower legs
  - Anosmia (lack of smell)
  - Lack of taste
  - None
  - Other (please specify)

3. “Did you later develop symptoms during your admission for delivery, that you did not initially have when you first came to labor and delivery?

YES ____ No____ Unsure ____

**If YES or UNSURE** 🡪

“Which symptoms did you later develop during your hospitalization?” (Select all endorsed)

- - Cough
  - Nasal congestion
  - Sore throat
  - Headache
  - Ear ache / ear congestion
  - Myalgias / body aches
  - Fever > 100F
  - Nausea
  - Vomiting
  - Diarrhea
  - Vision changes
  - Rash
  - SOB/dyspnea at rest
  - SOB/dyspnea with exertion
  - Chest pain or tightness
  - Abdominal pain (not incision pain)
  - Facial swelling
  - Pain / redness in lower legs
  - Anosmia (lack of smell)
  - Lack of taste
  - None
  - Other (please specify)

4. “Do you currently have symptoms concerning for Coronavirus?”

YES ____ No____ Unsure ____

**If YES or UNSURE** 🡪

“Which symptoms are you currently experiencing?” (Select all endorsed)

- - Cough
  - Nasal congestion
  - Sore throat
  - Headache
  - Ear ache / ear congestion
  - Myalgias / body aches
  - Fever > 100F (If >101**)
  - Nausea
  - Vomiting
  - Diarrhea
  - Vision changes**
  - Rash
  - SOB/dyspnea at rest**
  - SOB/dyspnea with exertion**
  - Chest pain or tightness**
  - Abdominal pain (not incision)
  - Facial swelling
  - Pain / redness in lower legs**
  - Anosmia (lack of smell)
  - Lack of taste
  - None
  - Other (please specify)

***** Refer patient for medical evaluation***

5. If answering YES to ANY symptoms, please ask🡪

“When did these symptoms first start?” (SPECIFY FOR EACH SYMPTOM – THE NUMBER OF DAYS AFTER DISCHARGE THAT THEY STARTED)

- - Cough_____­­­_____ Nasal congestion _____­­­_____
  - Sore throat_____­­­_____ Headache_____­­­_____
  - Ear ache / ear congestion _____­­­_____ Myalgias / body aches_____­­­_____
  - Fever > 100F Nausea_____­­­_____
  - Vomiting_____­­­_____ Diarrhea_____­­­_____
  - Vision changes_____­­­_____ Rash_____­­­_____
  - SOB/dyspnea at rest_____­­­_____ SOB/dyspnea with exertion_____­­­_____
  - Chest pain or tightness_____­­­_____ Abdominal pain (not incision)_____­­­___
  - Facial swelling_____­­­_____ Pain / redness in lower legs_____­­­_____
  - Anosmia (lack of smell)_____­­­_____ Lack of taste_____­­­_____
  - Other (please specify) _____­­­_____

6. “How many days did each of these symptoms last?” (SPECIFY FOR EACH SYMPTOM – THE NUMBER OF DAYS THAT THEY LASTED, or if STILL experiencing)

- - Cough_____­­­_____ Nasal congestion _____­­­_____
  - Sore throat_____­­­_____ Headache_____­­­_____
  - Ear ache / ear congestion _____­­­____ Myalgias / body aches_____­­­_____
  - Fever > 100F Nausea_____­­­_____
  - Vomiting_____­­­_____ Diarrhea_____­­­_____
  - Vision changes_____­­­_____ Rash_____­­­_____
  - SOB/dyspnea at rest_____­­­_____ SOB/dyspnea with exertion_____­­­_____
  - Chest pain or tightness_____­­­_____ Abdominal pain (not incision)_____­­­___
  - Facial swelling_____­­­_____ Pain / redness in lower legs_____­­­_____
  - Anosmia (lack of smell)_____­­­_____ Lack of taste_____­­­_____
  - Other (please specify) _____­­­_____

7. “How many days ago did symptoms stop?” ________

8. “Have you had any fevers since being discharged home?” Yes / No / Unsure

9. “What was the highest temperature you had since returning home?” ________ F/C

10. “When did you have this highest temperature?” (Number of days after discharge) _________

11. “Since you were discharged home, what medications are you taking, or have you taken for pain relief or relief or for fever?” (Select all that apply)

- NSAIDS (Ibuprofen, Motrin, Aleve, Advil, etc)
- Tylenol
- Dilaudid
- Oxycodone / Hydrocodone
- Tramadol
- Other
- None

12. “Did you receive any treatment / medications for Coronavirus?” Yes / No

**If YES** 🡪 “Which of the following medications did you receive?” (Select all that apply)

- Plaquenil / Hydroxychloroquine
- Azithromycin
- Remdesivir
- Plasma treatment
- Blood thinning medication (Heparin or lovenox)
- Keflex
- Other antibiotic
- Unsure
- Other medication_________________________
- None

13. “Since you were discharged after delivery, have you required evaluation in ER or any other urgent care facility for Coronavirus related symptoms?” Yes / No

**If YES** 🡪

- “How many days after discharge home did you seek medical evaluation?” __________
- “Were you admitted to the hospital?” Yes/No
- “How many days did you require admission?” (overnight = 1 day) __________
- “Were you admitted to the ICU?” Yes / No
- “Did you require supplemental oxygen therapy?” Yes / No

**IF YES** 🡪 “Which of the following did you receive?” (Select all that apply)

- Nasal canula
- BiPAP
- CPAP
- NBR mask
- Unsure
- “Did you require mechanical ventilation or intubation?” Yes / No

14. “Are you currently breastfeeding or pumping?” (Select one)

- - Yes- only breastfeeding
  - Yes – breastfeeding and pumping
  - Yes – breast pumping only
  - Yes—but also supplementing with bottle
  - No - only bottle feeding

15. “Are you wearing a mask while you are breastfeeding” Yes / No / Sometimes

16. “How is your baby doing?” (Select one)

- - Good / healthy
  - He/she has been sick
  - He/she is in the NICU
  - Unsure
  - Other

17. “Has your baby shown any symptoms of COVID-19 or other respiratory illness?” Yes / No

18. “Has your baby required medical evaluation for any concerning symptoms?” Yes / No

19. “Has your baby tested positive for COVID 19?” Yes / No

**If YES** 🡪

- “How many days old was baby when diagnosed?” _______
- “Did your baby require hospitalization for COVID-19?” Yes / No

20. “Was your partner tested for Coronavirus when you were admitted to labor and delivery?” Yes/No

If YES🡪

- “Did your partner test positive for coronavirus?” Yes / No / Pending

21. Was your partner or a support person allowed to be present for birth? Yes / No

20. “Are you wearing a mask around others?” Yes / No / Sometimes

21.“Are you practicing social distancing at home?” Yes / No / Sometimes

22. Are you washing your hands before touching your baby?

- - Yes with soap and water
  - Yes with hand sanitizer
  - Yes, with soap and water and hand sanitizer
  - Sometimes
  - No

'Please make sure that you continue to take precautions.”

“I know I've asked many questions.”

“Do you have any questions for me?” “These are trying times and we are here for you. Stay safe.”

“If your partner is positive for coronavirus, I recommend he/she/they continue to take precautions and seek medical guidance from his/her/them health care provider.”
